# Supplementary material for: Assessing geographical and economic inequalities in caesarean section rates between the districts of Bihar, India: a secondary analysis of the National Family Health Survey
Source: BMJ Open. 2022 Jan 5;12(1):e055326. doi: 10.1136/bmjopen-2021-055326 (PMC8739431; doi:10.1136/bmjopen-2021-055326)
Supplement: Author's manuscript [file bmjopen-2021-055326.draft_revisions.pdf]

BMJ Open is committed to open peer review. As part of this commitment we make the peer review history of every article we publish publicly available.

When an article is published we post the peer reviewers' comments and the authors' responses online. We also post the versions of the paper that were used during peer review. These are the versions that the peer review comments apply to.

The versions of the paper that follow are the versions that were submitted during the peer review process. They are not the versions of record or the final published versions. They should not be cited or distributed as the published version of this manuscript.

BMJ Open is an open access journal and the full, final, typeset and author-corrected version of record of the manuscript is available on our site with no access controls, subscription charges or pay-per-view fees (http://bmjopen.bmj.com).

If you have any questions on BMJ Open's open peer review process please email info.bmjopen@bmj.com

BMJ Open

From Unmet Need To Inequality: Caesarean Section Across Wealth Quintiles In Bihar, India

Journal:	*BMJ Open*
Manuscript ID	bmjopen-2021-055326
Article Type:	Original research
Date Submitted by the Author:	13-Jul-2021
Complete List of Authors:	Shukla, Minal; WHO Collaboration Centre for Research in Surgical Care Delivery in LMICs<br>Mohan, Monali; WHO Collaboration Centre for Research in Surgical Care Delivery in LMICs, Mumbai, India,<br>van Duinen, Alex; NTNU Fakultet for ingeniorvitenskap og teknologi Trondheim, Faculty of Medicine and Health Sciences Department of Cancer Research and Molecular Medicine; Sankt Olavs Hospital Universitetssykehuset i Trondheim, Department of Surgery<br>Gadgil, Anita; World Health Organization Collaborating Center for Research in Surgical Care Delivery in Low-and-Middle Income Countries<br>Bakker, Juul; Médecins Sans Frontières, Amsterdam, Netherlands<br>Bhushan, Pranav ; Ministry of health and family welfare, GoI<br>Roy, Nobhojit; Karolinska Institutet, Department of Global Public Health
Keywords:	OBSTETRICS, EPIDEMIOLOGY, Health policy < HEALTH SERVICES ADMINISTRATION & MANAGEMENT

SCHOLARONE™
Manuscripts

I, the Submitting Author has the right to grant and does grant on behalf of all authors of the Work (as defined in the below author licence), an exclusive licence and/or a non-exclusive licence for contributions from authors who are: i) UK Crown employees; ii) where BMJ has agreed a CC-BY licence shall apply, and/or iii) in accordance with the terms applicable for US Federal Government officers or employees acting as part of their official duties; on a worldwide, perpetual, irrevocable, royalty-free basis to BMJ Publishing Group Ltd ("BMJ") its licensees and where the relevant Journal is co-owned by BMJ to the co-owners of the Journal, to publish the Work in this journal and any other BMJ products and to exploit all rights, as set out in our licence.

The Submitting Author accepts and understands that any supply made under these terms is made by BMJ to the Submitting Author unless you are acting as an employee on behalf of your employer or a postgraduate student of an affiliated institution which is paying any applicable article publishing charge ("APC") for Open Access articles. Where the Submitting Author wishes to make the Work available on an Open Access basis (and intends to pay the relevant APC), the terms of reuse of such Open Access shall be governed by a Creative Commons licence – details of these licences and which Creative Commons licence will apply to this Work are set out in our licence referred to above.

Other than as permitted in any relevant BMJ Author's Self Archiving Policies, I confirm this Work has not been accepted for publication elsewhere, is not being considered for publication elsewhere and does not duplicate material already published. I confirm all authors consent to publication of this Work and authorise the granting of this licence.

For peer review only

Title page:

Title: From Unmet Need To Inequality: Caesarean Section Across Wealth Quintiles In Bihar, India.

Corresponding author.

Dr Nobhojit Roy: Address: 601, Balaji Apartment, Nerul sec 15, Navi Mumbai, Maharashtra India. Email ID: nobhojit.roy@ki.se; Mobile Number: +91 98212 91225

Authors

1. Minal Shukla, Global Public Health Expert WHO Collaboration Centre for Research in Surgical Care Delivery in LMICs, Mumbai, India,, shukla.minal@gmail.com
2. Monali Mohan, Consultant, WHO Collaboration Centre for Research in Surgical Care Delivery in LMICs, Mumbai, India, monalimohangupta@gmail.com.
3. Alex Van Duinen

   1. Institute of Clinical and Molecular Medicine, Norwegian University of Science and Technology, Trondheim, Norway. alexvanduinen@gmail.com
4. Anita Gadgil, "Department of surgery, BARC Hospital, WHO Collaboration Centre for Reserach in Surgical Care Delivery in LMICs, Mumbai, India, anitgadgil@gmail.com
5. Juul Bakker, Médecins Sans Frontières, Amsterdam, Netherlands juulbakker@gmail.com
6. Pranav Bhushan, Senior Technical officer, Aspirational District Unit (ADU), Ministry of health and family welfare, GoI, New Delhi, Indiapranav.nru@gmail.com.
7. Nobhojit Roy, Department of Public Health Sciences, Karolinska institute, Stockholm, Sweden nobhojit.roy@ki.se

For peer review only - http://bmjopen.bmj.com/site/about/guidelines.xhtml

Keywords: Health Equity, Caesarean Section, LMIC, Maternal Health Services, Socioeconomic Factors

-**Word count:** 3486.

Title: From Unmet Need To Inequality: Caesarean Section Across Wealth Quintiles In Bihar, India.

Abstract

Background: In India caesarean section rates are now almost at par with the global figures. In Bihar, which is most populous and poorest states in India caesarean sections increased over the last decade but an optimum aggregate caesarean section rate at the state level may conceal inequities at the district level.

Objectives: The primary aim of this study is to analyse the inequalities in the geographic and socioeconomic distribution of ‘caesarean sections between the districts of Bihar. The secondary aim was to compare the contribution of free-for-service Government funded public facilities and fee-for-service private facilities.

Setting: Bihar, with a population in the 2011 census of approximately 104 million people, has a low GDP per capita (US$610) compared to other Indian states. The state has the highest crude birth rate (26.1 per 1,000 population) in India, with one baby born every two seconds. Bihar is divided into 38 administrative districts, 101 subdivisions and 534 blocks. Each district has a district (Sadar) hospital, and six districts also have one or more medical college hospitals.

Methods This retrospective secondary data analysis was based on open source national datasets from ‘National Family Health Surveys’ of last two rounds.

Participants: Secondary data analysis of pregnant women delivering in public and private institutions.

Results: Caesarean section rate increased from 6.20% in 2015 to 9.70% in 2019 in Bihar.

For peer review only - http://bmjopen.bmj.com/site/about/guidelines.xhtml

Districts with a lower proportion of poor had higher caesarean section rates (R^2=0.45) among all institutional births with 10.32% among them in private and 2.89% in public facilities. Access to private caesarean sections decreased (R^2=0.46) for districts with poorer populations.

Conclusion: There are marked inequalities in access to caesarean sections. Public sector need to strengthen to maintain access to obstetric services for those that need it the most.

Article summary

- Strength of this study is it used the secondary data from India's largest and most reliable household-level survey data sources.NFHS.
- Limitations of study is NFHS -4 used to determine wealth quintile data because NFHS-5 has not yet released its wealth quintile data at the time of writing this paper.
- Evidence is mixed on optimum caesarean section in population to have maximum impact on reducing maternal mortality, the disaggregated cut-offs of 10% and 20% for adequate caesarean section rates in population were considered practical.

Introduction

In many low- and middle-income countries (LMICs), poor access to life-saving caesarean sections is a risk factor for maternal death. The caesarean section rate serves as a proxy measure of the availability of comprehensive emergency obstetric and newborn care (CEmONC) services. A rate below 5% usually denotes a lack of access to CEmONC services. Though the 'optimum' caesarean section rate remains elusive, rates >20% are not associated with any further reduction in maternal deaths. (1–3)

In India, the national caesarean section rate is climbing and almost at par with global figures. Further, the maternal mortality ratio in India has decreased from 437 to 113 in a quarter of a century. (4) Government programs in India have contributed to increasing institutional deliveries, but the private sector has a strong role in increasing caesarean

For peer review only - http://bmjopen.bmj.com/site/about/guidelines.xhtml

section rates. In addition, government maternal health programs, strong advocacy, and improved infrastructure and staffing have driven the increase in caesarean section rates in LMICs. (5)

Bihar, a relatively poor state in East India bordering Nepal, has seen an increase in the caesarean section rate from 6.20% in 2015 to 9.70% in 2019. (6,7) As this figure approaches 10%, it may suggest that the population's need for caesarean sections is met. Given the prevalent social inequalities, an optimum aggregate caesarean section rate at the state level may conceal inequities at the district level. Aggregated statistics conceal women who need but do not receive a caesarean section due to access or financial barriers by compensating with those who receive a caesarean section not medically indicated. Therefore, a more granular analysis of caesarean section rates at the district level is needed. (8)

In this paper, we report differences in caesarean section rates between population subgroups based on socioeconomic status across district wealth quintiles in Bihar. The study aimed to assess the geographical and economic inequalities in caesarean section rates between the districts of Bihar. In addition, the contributions of free government-funded public facilities and fee-for-service private facilities to population caesarean section rates are compared.

Methods

We conducted a secondary analysis of publicly available data from the National Family Health Survey (NFHS), a large-scale multi-round survey conducted in a representative sample of households throughout India. (6,9) The NFHS provides information on health and nutrition indicators at the sub-national level. The Government of India Ministry of Health and Family Welfare selected the International Institute for Population Sciences,

For peer review only - http://bmjopen.bmj.com/site/about/guidelines.xhtml

Mumbai, as the nodal agency for the various rounds. The Woman's Schedule covered a wide variety of topics, including women's characteristics, marriage, fertility, contraception, children's immunisations and healthcare, nutrition, reproductive health, sexual behaviour, HIV/AIDS, women's empowerment and domestic violence, but not maternal deaths.

Data sources

For Bihar, the Cross -5 collected information in 2019–2020 from 35,834 households, which included 42,843 women aged 15–49 years. (6) The NFHS records whether a caesarean section was performed in a public or private facility. The wealth quintile data were available in the 2015–2016 NFHS-4 (6,9), and raw data for Bihar were provided by the nodal agency on request.

Study setting

Bihar, with a population in the 2011 census of approximately 104 million people (10), has a low GDP per capita (US$610) compared to other Indian states. (8,11) In total, 88% of the population of Bihar lives in rural areas. The state has the highest crude birth rate (26.1 per 1,000 population) in India, with one baby born every two seconds. (6) Bihar is divided into 38 administrative districts, 101 subdivisions and 534 blocks. Each district has a district (Sadar) hospital, and six districts also have one or more medical college hospitals.

Bihar's government healthcare system functions on three levels. At the primary level, sub-health centres and primary health centres function as the first point of contact. These primary facilities, providing basic emergency obstetric and newborn care (BEmONC) services, are mostly staffed by nurses, general physicians and alternative medicine practitioners but have no surgical capacity. At the secondary level, 149 first referral units

For peer review only - http://bmjopen.bmj.com/site/about/guidelines.xhtml

(FRUs) function as first-level CEmONC units where caesarean sections are performed. These FRUs include district and sub-district hospitals (serving a population of 500,000–600,000) and community health centres (block-level hospitals), as per Indian Public Health Standards. (12) In addition, seven government and two autonomous medical colleges in Bihar are designated as tertiary centres, four of which are in the capital city of Patna (two government medical colleges and two autonomous medical colleges). Geographically, four medical colleges are located in North Bihar, four in Patna and only one in South Bihar. Medical colleges (though tertiary) fill a gap by also functioning as secondary health facilities because FRUs are poorly functional.

According to the NFHS-4 (7), the Indian population is divided into wealth quintiles. Based on this division, the proportion in each of these wealth quintiles for each district has been published. In Bihar, 80.23% of the population belongs to the two poorest quintiles, 11.41% to the middle quintile and only 8.36% to the two richest quintiles. (7) To compare districts, we combined the proportion of people in the two poorest quintiles.

Data analysis

Caesarean section rates and the proportion of institutional deliveries for each district were extracted from the NFHS-5, including the contributions by the public and private sectors. Since no optimum rate exists, we stratified the rates into <10%, 10–20% and >20% to measure equity in access to caesarean sections, from possible inadequate access to overuse. (13) The NFHS-4 and NFHS-5 caesarean section rates, respectively for 2015–2016 and 2019–2020, are presented in Figure 1 to demonstrate the geographical differences in change in rates between districts. Regression analysis was performed assessing the correlation between the proportion of the population in the two poorest quintiles and caesarean section rates. Both the caesarean section rate among all

For peer review only - http://bmjopen.bmj.com/site/about/guidelines.xhtml

institutional births and the proportion of institutional deliveries in private and public health facilities were considered using the extrapolated caesarean section rate from the NFHS-5. (6)

Patient and Public Involvement:

This is an analysis of the secondary data in public domain; collected in National Family Health Survey, rounds 4 and 5 in India. We have not done direct patient recruitment or any intervention on the participants as individual patient data was not used and the reports of these surveys and various open source data were analyzed. The results of the study will be published as open access manuscript in peer reviewed journal accessible to any researchers or patients.

Results

Caesarean section rate trends across geographical districts in Bihar

In Bihar, the caesarean section rate has increased from 6.20% in 2015 (NFHS-4) to 9.70% in 2019 (NFHS-5). Of the rate in 2019, 2.12% came from the public sector and 7.96% from the private sector. The NFHS-5 documented that the percentage of all live births delivered by caesarean section in public health facilities and private facilities was 3.60% and 39.60%, respectively.

Figure 1 shows the district-wise change in the rate of caesarean sections from 2015 to 2019. The number of districts with caesarean section rates below 10% decreased from 34 in 2015 to 21 in 2019. Using the 10% rate to evaluate access to caesarean sections in the 38 district populations, 14 districts were above 10% in 2019, three at 10% and 21 below 10%. Three districts (Jehanabad, Munger and Patna) had caesarean section rates above 20%.

For peer review only

The degree of change in caesarean section rates was uneven at the district level. Of the 38 districts, 28 demonstrated a remarkable increase of 5.90% in caesarean section rates in four years (from 5.5% in 2015 to 11.41% in 2019). However, against this trend was a 2.10% reduction in caesarean section rates in 10 districts (from 8.49% in 2015 to 6.49% in 2019).

Surrounding Patna are 10 adjacent districts within 86 kilometres or a two-hour driving time. In these districts, 77.44% of the population belong to the two poorest wealth quintiles. Moving away from the capital city, we noted a decrease in the caesarean section rate to 11.32% (of which 9.34% was in private and 1.98% in public facilities). The more remote districts are on average 192 kilometres away from the capital, or 5–6 hours transit. Of the population in these remote districts, 81.09% are in the two poorest quintiles, and the furthest from the capital had a further caesarean section rate drop to 9.70% (of which 7.53% was in private and 2.17% in public facilities). Public facilities conducted proportionally more caesarean sections among poorer populations than the richer quintiles.

Figure 1: Caesarean section rate by district in 2015 and 2019

Comparison of caesarean section rate in the districts of Bihar in 2015 (NFHS-4) and 2019 (NFHS-5).

Among all institutional births at public facilities, the caesarean section rate markedly decreased from 8.2% in 2015 to 2.1% in 2019. Contrary to the overall rising trend of caesarean section rate, the number of government public facilities performing at least 10% caesarean sections among all deliveries dropped from eight in 2015 to zero in 2015. The districts with university teaching hospitals and medical colleges (Bhagalpur,

For peer review only - http://bmjopen.bmj.com/site/about/guidelines.xhtml

Nalanda, Darbhanga, Gaya, Muzaffarpur and Patna) in addition to district hospitals had district-level caesarean section rates higher than 10%, except West Champaran (6.4%).

In contrast, in private facilities, the caesarean section rate increased from 6.79% in 2015 to 7.96% in 2019. Only four districts in 2015 had a caesarean section rate of >10% in private facilities, which increased to nine districts in 2019.

Caesarean section rate trends by district wealth quintiles in Bihar

Districts with higher proportions of the two richest quintiles (Bhagalpur, Buxar, Nalanda, Patna and Rohtas) had the highest caesarean section rates among all institutional births, with 11.79% in private facilities and 2.68% in public health facilities. Patna, the capital district of Bihar (and with the wealthiest people in the state), had the highest caesarean section rate in private facilities (17.91%) and a higher than average rate in public health facilities (4.03%). Supaul is the poorest district in Bihar, with 94.09% of the population in the lowest two quintiles, and of all deliveries, 1.80% and 1.91% were delivered by caesarean respectively in private and public facilities.

With decreasing wealth was a corresponding decrease in caesarean section rates (Figure 2). The negative correlation (R^2 = 0.46) between population wealth quintiles and caesarean section rates in private health facilities demonstrates that access to private caesarean sections drops dramatically in poorer populations. However, caesarean section rates dropped in poorer populations even in public facilities, though the correlation was weak (R^2 = 0.10) Public health facilities function independent of the wealth status of the population and are proportionately more available for the poor, who have lesser access to private care.

For peer review only - http://bmjopen.bmj.com/site/about/guidelines.xhtml

Figure 2: Proportion of total deliveries by Caesarean section in private and public sector by wealth status in the districts of Bihar

Proportion of deliveries that are delivered by caesarean section in the public and private sector by wealth status in the districts of Bihar.

Figure 3: Proportion of total deliveries in private and public institutions by wealth status in the districts of Bihar.

Institutional deliveries by wealth status in the districts of Bihar, including the relative contribution of the public and private sector.

The institutional delivery rate in Bihar has increased from 63.80% in 2005 to 76.20% in 2019. Overall institutional births show a negative linear correlation ($R^2 = 0.43$) with the wealth status of the population, and homebirths are still associated with poorer populations. Populations in poorer districts are more reliant on public facilities for institutional deliveries and birth preparedness. With increasing poverty, institutional deliveries in private facilities decreased, with a negative linear correlation ($R^2 = 0.64$). Among the poorest two quintiles, the proportion of institutional deliveries in public facilities was higher than in private facilities. In the same group, the opposite was observed for caesarean sections, which are predominantly performed in the private sector.

Discussion

The caesarean section rate is driven by supply-side (such as resources within the health system, healthcare policy and strategies, health financing systems and perceptions of healthcare professionals) and demand-side (such as socioeconomic status, population preference and perceptions and trust in the health system) determinants. This study

For peer review only - http://bmjopen.bmj.com/site/about/guidelines.xhtml

reinforces the importance of government maternal health programs in promoting institutional deliveries in resource-poor economies. The poor population chooses to deliver in public health facilities because they are affordable and incentivised. In the poorest regions, establishing private facilities is not profitable because the paying capacity of the population is low, and therefore the private option is simply not available. The scale-up of institutional deliveries has been possible due to the extensive and prolonged training programs for auxiliary nurses and general nurse midwives, supported by task-shifted cadres of helpers in the labour delivery suite. (14)

Uneven caesarean section rate across geographical distribution

Bihar is divided into North and South Bihar, with marked variations in geography and health. (15) North Bihar is downstream of the seasonally flooding Himalayan rivers, whereas South Bihar is in the drought zone. In both areas, the availability and utilisation of health service facilities are driven by the socioeconomic status of the population and provider factors rather than physical access. (14) Geographical access to health facilities has improved in Bihar, with better roads, electricity and transportation over the past decade. (9) The eastern districts in Bihar are insecure workplaces for healthcare professionals due to political instability. Bihar has the lowest population-to-doctor ratio in the country, and the available doctors tend to choose urban areas for government service or private practice. (16)

The caesarean section rate varies across the districts of Bihar. We found that districts closer to the capital city of Patna had higher caesarean section rates. Other factors influencing the caesarean section rate were the number of active specialists, availability of private facilities and blood availability. All these factors decrease with distance from the capital. The caesarean section rates in Bihar display the entire spectrum from too little

For peer review only - http://bmjopen.bmj.com/site/about/guidelines.xhtml

(inadequate access) to too much (overuse), all within one state. The powerful global acceptance of technological solutions and medicalising birth is set against the local traditional preference for vaginal delivery, regardless of educational status and across wealth quintiles in the Bihar population. The poorer population faces affordability and access barriers to caesarean sections. (16) Cultural beliefs and practices about vaginal birth, irrespective of wealth quintiles, make it the preferred mode of delivery over caesarean section. Performing a caesarean section is still viewed as a lack of competence of the provider to perform an unassisted or assisted vaginal delivery, but this may also simply reflect a lack of trust or fear of a surgical procedure. Demand for caesarean sections has been relatively uncommon in publications from this state (14,16–19) due to cosmetic concerns, fear of pain during childbirth and sexual functioning concerns foregrounded by the upper wealth quintiles from other nations (2) or other Indian states (17).

Distribution of caesarean section rates between public and private health facilities

Free-service public facilities are the first point of access for women with high-risk pregnancies in this predominantly poor state. However, while BEmONC services have been strengthened in public facilities, our findings show a quantitative reduction in CEmONC services available to the same women who chose to deliver in public facilities. While more than six out of 10 women delivered in public facilities, only around one in 28 women (3.6%) received a caesarean section in these public hospitals. Three in five children were delivered by caesarean section in private hospitals (39.6%). A proportionate decline has occurred in the outputs and performance of public health facilities in Bihar over five years (2015–2019). Of 149 designated public FRUs, only 40 provide some CEmONC services, usually during office hours. The better-functioning public facilities, with significantly higher caesarean section rates, are attributed to the

For peer review only - http://bmjopen.bmj.com/site/about/guidelines.xhtml

presence of medical colleges, teams of specialist doctors and greater urbanisation in those districts. (2) In the poorer-performing districts, low output is commonly attributed to inadequate infrastructure and staffing. However, rates remain low even in well-resourced public facilities with adequate infrastructure. (20) Our results show that caesarean section services for poorer and the poorest populations are available in neither public facilities nor private health facilities.

The supply-side provider barriers in public facilities are hesitancy to treat at-risk pregnant women, lack of clinical support for complications, administrative support for litigation, and occasional episodes of violence against healthcare workers. Government doctors who refuse to perform caesarean sections in public facilities are willing to operate on the same high-risk women in private facilities. Professional social networks provide a safety net and allow for higher risk-taking in private facilities, though the infrastructure is better in public facilities. Financial incentives to providers via government schemes (Janani Shishu Suraksha Karyakram) with performance-based incentives for doctors have not increased the provision of caesarean sections at government facilities. (15) Women with high-risk pregnancies are referred to higher-level facilities (private facilities, medical colleges or tertiary facilities) for caesarean sections. (21) The demand-side patient barriers for low uptake of caesarean sections include user fees, sociocultural beliefs around the value of the procedure, a longer stay in the hospital and a trust deficit about the safety of caesarean sections. Costs in private hospitals are at least two to four times those in public hospitals. (6,22) The fee structure in the private sector is not regulated by the government, and the private sector is therefore free to decide user fees. Currently, the predominantly urban private sector in Bihar is still inadequate to provide for the needs of the 80% of the population in the two poorest wealth quintiles, and therefore public

For peer review only

For peer review only - http://bmjopen.bmj.com/site/about/guidelines.xhtml

facilities will need to be functional to provide free and affordable caesarean section services.

Caesarean section distribution across wealth quintiles and equitable solutions

Caesarean section is a life-saving operation that should be available to all women. The probable reasons for the inequity and unaffordability of healthcare services include insufficient public financing and consequent out-of-pocket expenditure to meet the high costs of caesarean section services. (23)

To address inequity in access, the Government of India launched a conditional cash-transfer scheme to promote institutional deliveries by providing free services to neonates and pregnant women, aiming to encourage marginalised pregnant women to use free public health facilities. More recently, the world's largest health insurance scheme, Pradhan Mantri Jan Arogya Yojana (PM-JAY), was launched in 2018 to provide free services for the 500 million poor in India. Caesarean section is included in the PM-JAY maternal package to mitigate the impact of the catastrophic expenditure incurred. However, the overall uptake of PM-JAY reimbursement rates by private providers remains poor in the state. (24)

Lessons can be learned from other LMICs, such as Cambodia, Indonesia and Ghana, which have previously introduced policies to remove user fees for pregnant women. (2) Rwanda has set an example with its community-based health insurance scheme to reduce out-of-pocket payments for patients and a performance-based financing system to incentivise providers. (25) The key findings from our results – that although the overall caesarean rate has increased among the poor, the rates for the poorest subgroup still fall short – are also reflected by findings from Ghana and Rwanda. Like Ghana, Bihar exempts women from paying for delivery care and has a state policy of demand-side

For peer review only - http://bmjopen.bmj.com/site/about/guidelines.xhtml

financing by incentivising institutional deliveries to reduce barriers to access to quality care in remote areas. Cambodia has identified and targeted the poor for user fee exemptions and health insurance provision through health equity funds. This scheme is similar to Indonesia's social health insurance program, *Jamkesmas*, for poor and near-poor people, which has been shown to improve access and reduce financial barriers. (26) In Bhutan, no wealth-related inequality has been observed, with around one fifth of deliveries by caesarean section, regardless of wealth. However, no privatisation of healthcare exists, and there is only a 13% difference between the richest and the poorest in this LMIC. (2,27) Burundi, like Bihar, demonstrates increasing inequalities and caesarean section disparities with improved access to maternal healthcare. (28)

Conclusion

Aggregated state-level caesarean section rates conceal the inequity of inadequate access to emergency obstetric care among the poorest population, which is compensated by the high levels of caesarean use in the richest and most advantaged subgroups. Using relevant equity stratifiers, population subgroups and specific strategies need to be identified to address the inequities by improving access to maternal and newborn health services in public health facilities. The role of the private sector in meeting the demand for caesarean sections for the population is strongly substantiated, but the concomitant out-of-pocket expenditure is debilitating and catastrophic.

contributorship statement: Nobhojit Roy, Anita Gadgil and Alex van Duinen conceptualised and planned research project. Minal Shukla, Monali Mohan, Juul Bakker and Pranav Bhushan conducted the analysis. All supported in writing the manuscript.

For peer review only - http://bmjopen.bmj.com/site/about/guidelines.xhtml

Competing interests: None of the authors have any competing interests.

Funding: It was a non-funded project.

Data sharing statement: Data are available in a public, open access repository, additionally, data can be shared by the corresponding author.

Ethics approval statement: This was an analysis of secondary data from open access and no primary data has been collected from human subjects.

References

1. Sobhy S, Arroyo-Manzano D, Murugesu N, Karthikeyan G, Kumar V, Kaur I, et al. Maternal and perinatal mortality and complications associated with caesarean section in low-income and middle-income countries: a systematic review and meta-analysis. Lancet [Internet]. 2019;393(10184):1973–82. Available from: http://dx.doi.org/10.1016/S0140-6736(18)32386-9

2. Boatin AA, Schlotheuber A, Betran AP, Moller AB, Barros AJD, Boerma T, et al. Within country inequalities in caesarean section rates: Observational study of 72 low and middle income countries. BMJ. 2018;360.

3. Molina G, Weiser TG, Lipsitz SR, Esquivel MM, Uribe-Leitz T, Azad T, et al. Relationship between cesarean delivery rate and maternal and neonatal mortality. JAMA - J Am Med Assoc. 2015;314(21):2263–70.

4. Sample Registration Survey: MMR 2016-2018; SRS Bulletin 2018 Vol 53 No.1 [Internet]. New Delhi; 2020. Available from: https://censusindia.gov.in/vital_statistics/SRS_Bulletins/SRS Bulletin_2018.pdf

5. Meara JG, Leather AJM, Hagander L, Alkire BC, Alonso N, Ameh EA, et al. Global Surgery 2030: evidence and solutions for achieving health, welfare, and economic

For peer review only - http://bmjopen.bmj.com/site/about/guidelines.xhtml

development. Lancet [Internet]. 2015;386(9993):569–624. Available from: http://www.ncbi.nlm.nih.gov/pubmed/25924834?otool=karolib&tool=karolinska

6. International Institute for Population Sciences. National Family Health Survey-5 (Bihar State Report) 2019-2020 [Internet]. Mumbai; 2020. Available from: http://rchiips.org/nfhs/NFHS-5_FCTS/FactSheet_BR.pdf

7. International Institute for Population Sciences. National Family Health Survey (NFHS-4) 2015-16 India. Int Inst Popul Sci ICF [Internet]. 2017;1–192. Available from: http://rchiips.org/NFHS/NFHS-4Reports/India.pdf

8. Guilmoto CZ, Dumont A. Trends, Regional Variations, and Socioeconomic Disparities in Cesarean Births in India, 2010-2016. JAMA Netw open. 2019;2(3):e190526.

9. Bihar. National Family Health Survey IV, Bihar [Internet]. Ministry of Health and Family Welfare, Government of India. 2017. p. 1–170. Available from: http://rchiips.org/nfhs/NFHS-4Reports/Bihar.pdf

10. Census of India 2011: Bihar [Internet]. [cited 2020 Jul 20]. Available from: http://www.census2011.co.in/census/state/bihar.html

11. Srivastava S, Chaurasia H, Kumar Singh KJ, Chaudhary P. Exploring the spatial patterns of cesarean section delivery in India: Evidence from National Family Health Survey-4. Clin Epidemiol Glob Heal [Internet]. 2019;(September):1–9. Available from: https://doi.org/10.1016/j.cegh.2019.09.012

12. Indian Public Health Standards (IPHS) Guidelines for District Hospital , Sub-District/Sub-Divisional Hospitals and CHC [Internet]. New Delhi; 2012. Available from: http://nhm.gov.in/images/pdf/guidelines/iphs/iphs-revised-guidlines-2012/community-health-centres.pdf

For peer review only

For peer review only - http://bmjopen.bmj.com/site/about/guidelines.xhtml

13. Betran AP, Torloni MR, Zhang JJ, Gülmezoglu AM. WHO statement on caesarean section rates. BJOG An Int J Obstet Gynaecol. 2016;123(5):667–70.

14. Creanga AA, Srikantiah S, Mahapatra T, Das A, Sonthalia S, Ranjan Moharana P, et al. Statewide implementation of a quality improvement initiative for reproductive, maternal, newborn and child health and nutrition in Bihar, India. J Glob Health. 2020;10(2):1–13.

15. Salazar M, Vora K, Annerstedt KS, De Costa A. Caesarean sections in the in the context of the Chiranjeevi Yojana public private partnership program to promote institutional birth in Gujarat, India; Does the embedded disincentive for caesarean section work? Int J Equity Health. 2019;18(1):1–8.

16. Kaur J, Franzen SRP, Newton-Lewis T, Murphy G. Readiness of public health facilities to provide quality maternal and newborn care across the state of Bihar, India: A cross-sectional study of district hospitals and primary health centres. BMJ Open. 2019;9(7):1–10.

17. Vora KS, Yasobant S, Sengupta R, De Costa A, Upadhyay A, Mavalankar D V. Options for optimal coverage of free C section services for poor mothers in indian state of Gujarat: Location allocation analysis using GIS. PLoS One. 2015;10(9):1–16.

18. Office of the Registrar General & Census Commissioner. Annual Health Survey:2011-12 Fact Sheet Bihar. 2012;1–176. Available from: www.censusindia.gov.in

19. Dhirar N, Kumar R, Dudeja S. Upward trend of caesarean sections in India: Incision over precision. Vol. 31, National Medical Journal of India. 2018. p. 316–7.

20. 12th Common Review Mission [Internet]. New Delhi; 2019 [cited 2021 May 2]. Available from: http://nhsrcindia.org/sites/default/files/12th Common Review Mission-

For peer review only

Report 2018.pdf

21. Bhatia M, Banerjee K, Dixit P, Dwivedi LK. Assessment of Variation in Cesarean Delivery Rates Between Public and Private Health Facilities in India From 2005 to 2016. JAMA Netw open. 2020;3(8):e2015022.

22. Radhakrishnan A, Vasanthakumari K, PK B. Increasing Trend of Caesarean Rates in India: Evidence from NFHS-4. J Med Sci Clin Res [Internet]. 2017 Aug 7;5(8):26167–76. Available from: http://jmscr.igmpublication.org/v5-i8/31 jmscr.pdf

23. Balarajan Y, Selvaraj S, Subramanian S. Health care and equity in India. Lancet [Internet]. 2011 Feb;377(9764):505–15. Available from: https://linkinghub.elsevier.com/retrieve/pii/S0140673610618946

24. Report on Top Utilized Private Hospitals - Ayushman Bharat- Pradhan Mantri Jan Arogya Yojana (AB PM-JAY) [Internet]. New Delhi; 2020. Available from: https://pmjay.gov.in/sites/default/files/2019-10/Report on top utilized pvt hospitals_Final.pdf

25. Gibbons L, Belizán J, Lauer J, Betrán A, Merialdi M, Althabe F. The Global Numbers and Costs of Additionally Needed and Unnecessary Caesarean Sections Performed per Year: Overuse as a Barrier to Universal Coverage HEALTH SYSTEMS FINANCING [Internet]. Vol. 30, World Health Report 2010. 2010 Jan. Available from: https://www.researchgate.net/publication/265064468_The_Global_Numbers_and_Costs_of_Additionally_Needed_and_Unnecessary_Caesarean_Sections_Performed_per_Year_Overuse_as_a_Barrier_to_Universal_Coverage_HEALTH_SYSTEMS_FINANCING

26. Zahroh RI, Disney G, Betrán AP, Bohren MA. Trends and sociodemographic

For peer review only

For peer review only - http://bmjopen.bmj.com/site/about/guidelines.xhtml

inequalities in the use of caesarean section in Indonesia, 1987-2017. BMJ Glob Heal. 2020;5(12).

27. Hanson C, Betrán AP, Opondo C, Mkumbo E, Manzi F, Mbaruku G, et al. Trends in caesarean section rates between 2007 and 2013 in obstetric risk groups inspired by the Robson classification: results from population-based surveys in a low-resource setting. BJOG An Int J Obstet Gynaecol. 2019;126(6):690–700.

28. Yaya S, Zegeye B, Idriss-Wheeler D, Shibre G. Inequalities in caesarean section in Burundi: Evidence from the Burundi Demographic and Health Surveys (2010-2016). BMC Health Serv Res. 2020;20(1):1–8.

For peer review only - http://bmjopen.bmj.com/site/about/guidelines.xhtml

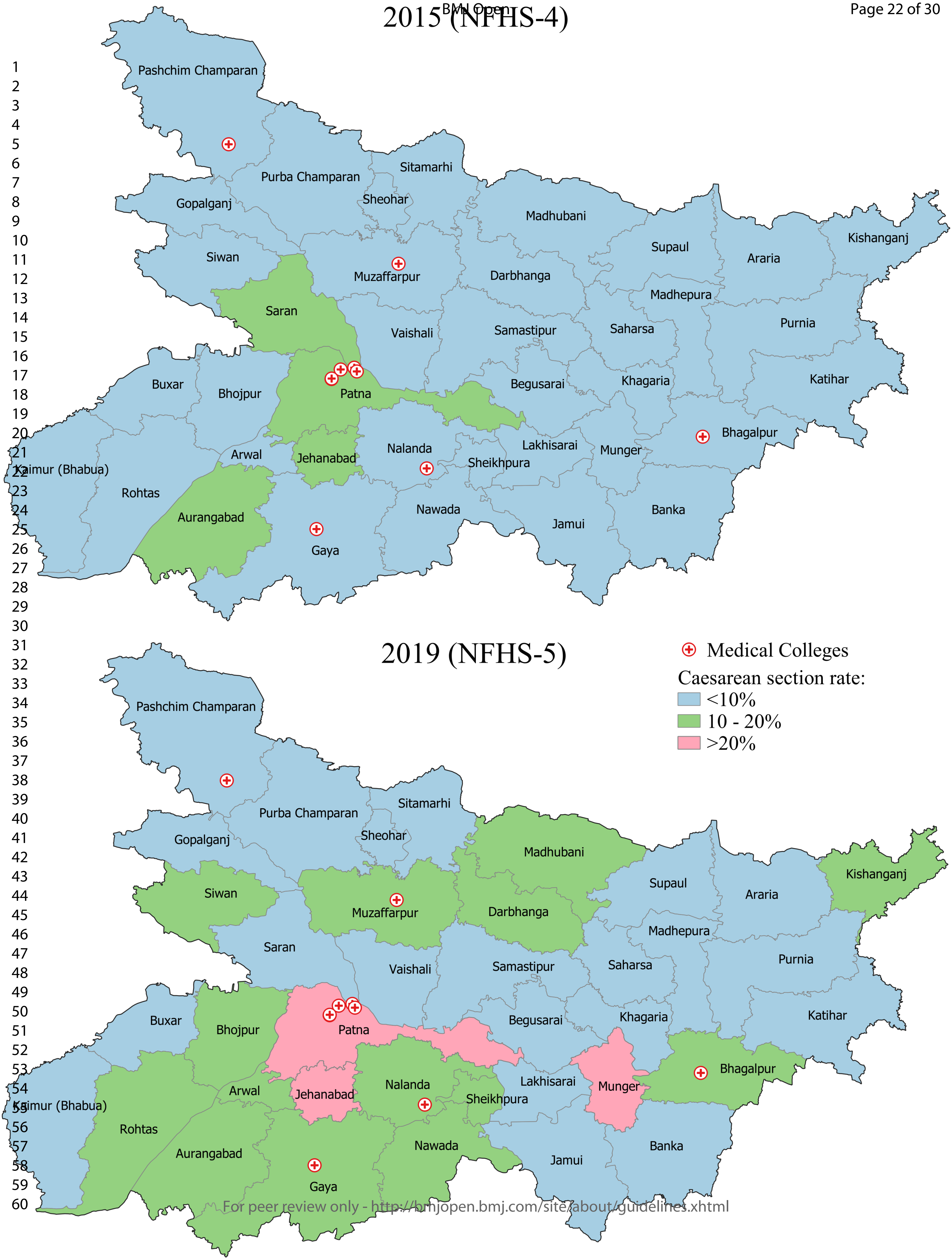
2015 (NFHS-4)
Pashchim Champaran
Purba Champaran
Sitamarhi
Sheohar
Gopalganj
Madhubani
Kishanganj
Supaul
Araria
Siwan
Muzaffarpur
Darbhanga
Madhepura
Saran
Purnia
Vaishali
Samastipur
Saharsa
Buxar
Bhojpur
Patna
Begusarai
Khagaria
Katihar
Bhagalpur
Lakhisarai
Munger
Arwal
Jehanabad
Nalanda
Sheikhpura
Kaimur (Bhabua)
Rohtas
Aurangabad
Nawada
Banka
Jamui
Gaya
2019 (NFHS-5)
Medical Colleges
Caesarean section rate:
<10%
10 - 20%
>20%
Pashchim Champaran
Purba Champaran
Sitamarhi
Sheohar
Gopalganj
Madhubani
Kishanganj
Supaul
Araria
Siwan
Muzaffarpur
Darbhanga
Madhepura
Saran
Purnia
Vaishali
Samastipur
Saharsa
Buxar
Bhojpur
Patna
Begusarai
Khagaria
Katihar
Bhagalpur
Lakhisarai
Munger
Arwal
Jehanabad
Nalanda
Sheikhpura
Kaimur (Bhabua)
Rohtas
Aurangabad
Nawada
Banka
Jamui
Gaya

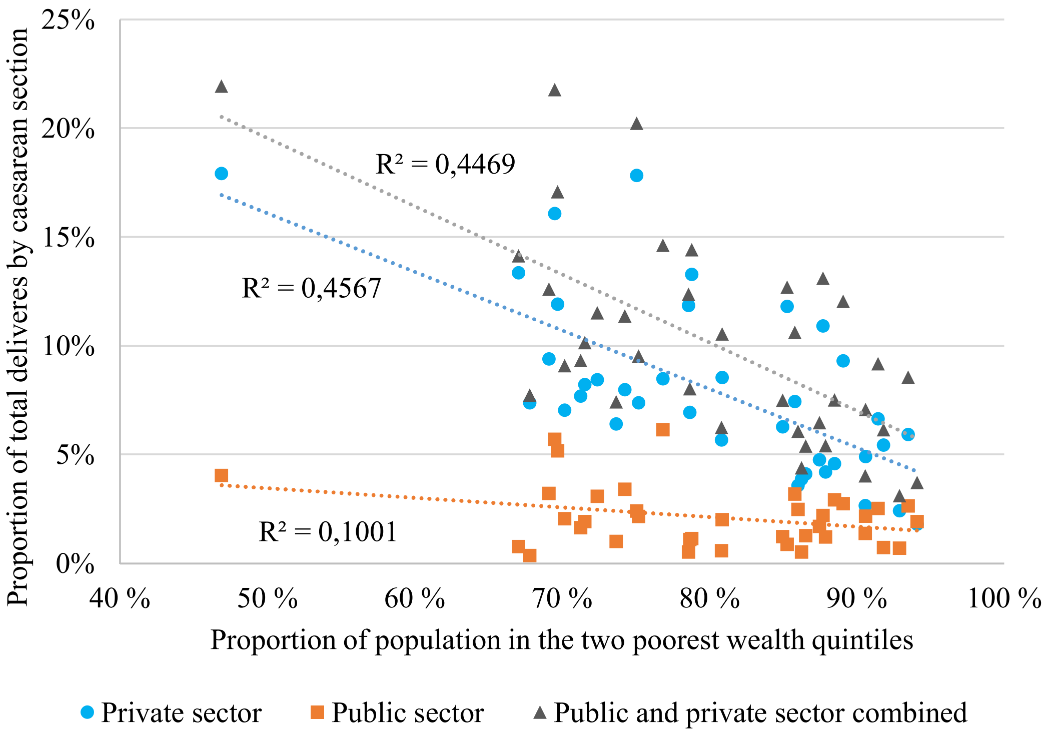

For peer review only

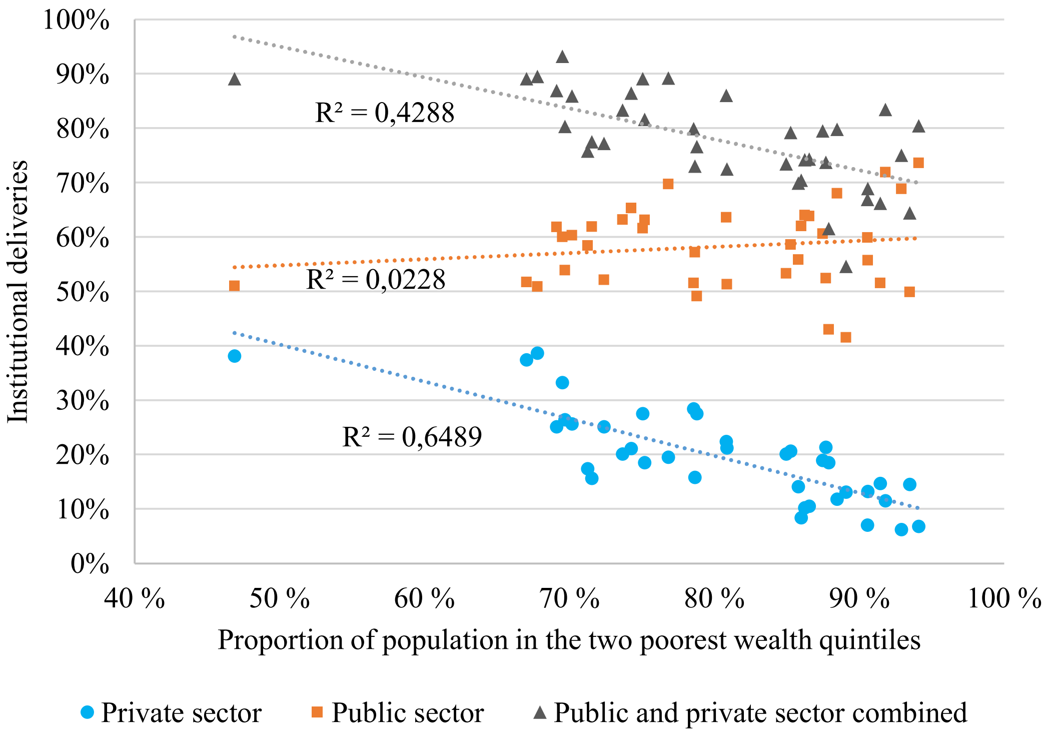

For peer review only

STROBE guidelines		
**Section/topic**	**Item number**	**Recommendation**
**Title and abstract**	1	From unmet need to inequality: Caesarean section across wealth quintiles in Bihar, India<br>Background: In India caesarean section rates are now almost at par with the global figures. In Bihar, which is most populous and poorest states in India caesarean sections increased over the last decade. Though state level aggregate caesarean rates in Bihar have risen to be near global figures, it conceals the inequities at the district level and vary as per economic status of the communities. The primary aim of this study is to analyse the inequalities in the geographic and socioeconomic distribution of 'caesarean sections between the districts of Bihar. The secondary aim was to compare the contribution of the free-for-service Government funded public facilities and fee-for-service private facilities.<br><br>Methods This retrospective secondary data analysis was based on open source national datasets from 'National Family Health Surveys' of last two rounds.<br><br>Results: Caesarean section rate increased from 6.20% in 2015 to 9.70% in 2019 in Bihar. Districts with a lower proportion of poor had higher caesarean section rates (R2=0.45) among all institutional births with 10.32% among them in private and 2.89% in public facilities. Access to private caesarean sections decreased (R2=0.46) for districts with poorer populations.

STROBE guidelines		
**Section/topic**	**Item number**	**Recommendation**
		Conclusion: There are marked inequalities in access to caesarean sections. Public sector need to strengthen to maintain access to obstetric services for those that need it the most.
**Introduction**		
Background/rationale	2	In India caesarean section rates are now almost at par with the global figures. In Bihar, which is most populous and poorest states in India caesarean sections increased over the last decade. Though state level aggregate caesarean rates in Bihar have risen to be near global figures, it conceals the inequities at the district level and vary as per economic status of the communities.
Objectives	3	The study aimed to assess the geographical and economic inequalities in caesarean section rates between the districts of Bihar. In addition, the contributions of free government-funded public facilities and fee-for-service private facilities to population caesarean section rates are compared.
**Methods**		

STROBE guidelines		
**Section/topic**	**Item number**	**Recommendation**
Study design	4	It is a Cross sectional study of secondary data study based on open source national datasets from ‘National Family Health Surveys’ of last two rounds in India (NFHS-4 (2015-16) and NFHS-5 (2019-20).
Setting	5	Study setting is the public health facilities and private facilities of northern state of India Bihar. It is a secondary data analysis from
Participants	6	Population covered in the two rounds of national survey from Bihar, India.
Variables	7	Total caesarean section rates, caesarean section rates in Private facilities and C section rates in public facilities in the districts of Bihar as available from the National survey . Wealth quintile data of the population covered in Bihar
Data sources/measurement	8*	This retrospective secondary data analysis was based on open source national datasets from ‘National Family Health Surveys’ of last two rounds.

STROBE guidelines		
**Section/topic**	**Item number**	**Recommendation**
Bias	9	The Government of India Ministry of Health and Family Welfare selected the International Institute for Population Sciences, Mumbai, as the nodal agency for the various rounds of this survey. Sample from Bihar gathered from 35,834 households, 42,483 women, and 4,897 men.
Study size	10	Entire data set collected from Bihar regarding caesarean section and distribution of population across wealth quintiles was used. No sample drawn from this
Quantitative variables	11	According to the NFHS-4 the Indian population is divided into wealth quintiles. Based on this division, the proportion in each of these wealth quintiles for each district has been published.
Statistical methods	12	Statistical methods used is regression analysis. Population sub-groups falling under two poorest wealth quintile pregnant women sought for delivery services in public and private health facilities.
Results		

STROBE guidelines		
**Section/topic**	**Item number**	**Recommendation**
Participants	13*	Entire data set collected from Bihar regarding caesarean section and distribution of population across wealth quintiles was used. No sample drawn from this
Descriptive data	14*	Not applicable
Outcome data	15*	Distribution of C sections across various wealth quintiles at districts in Bihar. Distribution of caesarean sections done in private and public facilities across wealth quintiles
Main results	16	Caesarean section rate increased from 6.20% in 2015 to 9.70% in 2019 in Bihar. Districts with a lower proportion of poor had higher caesarean section rates (R2=0.45) among all institutional births with 10.32% among them in private and 2.89% in public facilities. Access to private caesarean sections decreased (R2=0.46) for districts with poorer populations.
Other analyses	17	Not applicable.

STROBE guidelines		
**Section/topic**	**Item number**	**Recommendation**
Discussion		Across the LMIC inequality in health care access is evident because of various reasons like geography, economic conditions etc. Population sub group in poor wealth quintile agonize for access of services like institutional delivery and caesarean section in public as well as private health facilities. However government made many policy level decisions to reduce the hurdles for poor.
Key results	18	Caesarean section rate increased from 6.20% in 2015 to 9.70% in 2019 in Bihar. Districts with a lower proportion of poor had higher caesarean section rates (R2=0.45) among all institutional births with 10.32% among them in private and 2.89% in public facilities. Access to private caesarean sections decreased (R2=0.46) for districts with poorer populations.
Limitations	19	Limitations of study is NFHS -4 used to determine wealth quintile data. NFHS-5 has not yet released its wealth quintile data at the time of writing this paper. Evidence is mixed on what is the ideal rate of C section in population to have maximum impact on reducing maternal mortality, the disaggregated cut-offs of 10% and 20%for adequate c section rates in population were considered practical .

STROBE guidelines		
**Section/topic**	**Item number**	**Recommendation**
Interpretation	20	There are marked inequalities in access to caesarean sections. Public sector need to strengthen to maintain access to obstetric services for those that need it from poorer wealth quintiles
Generalizability	21	Not applicable.
Other information		
Funding	22	Non- funded research.

BMJ Open

Assessing geographical and economic inequalities in caesarean section rates between the districts of Bihar, India: A secondary analysis of the National Family Health Survey

Journal:	*BMJ Open*
Manuscript ID	bmjopen-2021-055326.R1
Article Type:	Original research
Date Submitted by the Author:	22-Sep-2021
Complete List of Authors:	Shukla, Minal; WHO Collaboration Centre for Research in Surgical Care Delivery in LMICs<br>Mohan, Monali; WHO Collaboration Centre for Research in Surgical Care Delivery in LMICs, Mumbai, India,<br>van Duinen, Alex; NTNU Fakultet for ingeniorvitenskap og teknologi Trondheim, Faculty of Medicine and Health Sciences Department of Cancer Research and Molecular Medicine; Sankt Olavs Hospital Universitetssykehuset i Trondheim, Department of Surgery<br>Gadgil, Anita; World Health Organization Collaborating Center for Research in Surgical Care Delivery in Low-and-Middle Income Countries<br>Bakker, Juul; Médecins Sans Frontières, Amsterdam, Netherlands<br>Bhushan, Pranav ; Ministry of health and family welfare, GoI<br>Roy, Nobhojit; Karolinska Institutet, Department of Global Public Health
**Primary Subject Heading**:	Health services research
Secondary Subject Heading:	Obstetrics and gynaecology, Surgery
Keywords:	OBSTETRICS, EPIDEMIOLOGY, Health policy < HEALTH SERVICES ADMINISTRATION & MANAGEMENT

SCHOLARONE™
Manuscripts

I, the Submitting Author has the right to grant and does grant on behalf of all authors of the Work (as defined in the below author licence), an exclusive licence and/or a non-exclusive licence for contributions from authors who are: i) UK Crown employees; ii) where BMJ has agreed a CC-BY licence shall apply, and/or iii) in accordance with the terms applicable for US Federal Government officers or employees acting as part of their official duties; on a worldwide, perpetual, irrevocable, royalty-free basis to BMJ Publishing Group Ltd ("BMJ") its licensees and where the relevant Journal is co-owned by BMJ to the co-owners of the Journal, to publish the Work in this journal and any other BMJ products and to exploit all rights, as set out in our licence.

The Submitting Author accepts and understands that any supply made under these terms is made by BMJ to the Submitting Author unless you are acting as an employee on behalf of your employer or a postgraduate student of an affiliated institution which is paying any applicable article publishing charge ("APC") for Open Access articles. Where the Submitting Author wishes to make the Work available on an Open Access basis (and intends to pay the relevant APC), the terms of reuse of such Open Access shall be governed by a Creative Commons licence – details of these licences and which Creative Commons licence will apply to this Work are set out in our licence referred to above.

Other than as permitted in any relevant BMJ Author's Self Archiving Policies, I confirm this Work has not been accepted for publication elsewhere, is not being considered for publication elsewhere and does not duplicate material already published. I confirm all authors consent to publication of this Work and authorise the granting of this licence.

For peer review only

Title page:

Title: Assessing geographical and economic inequalities in caesarean section rates between the districts of Bihar, India: A secondary analysis of the National Family Health Survey.

Corresponding author.

Dr Nobhojit Roy: Address: 601, Balaji Apartment, Nerul sec 15, Navi Mumbai, Maharashtra India. Email: nobhojit.roy@ki.se; Mobile Number: +91 98212 91225

Authors

1. Minal Shukla¶, Global Public Health Expert WHO Collaboration Centre for Research in Surgical Care Delivery in LMICs, Mumbai, India, shukla.minal@gmail.com.
2. Monali Mohan¶, Consultant, WHO Collaboration Centre for Research in Surgical Care Delivery in LMICs, Mumbai, India, monalimohangupta@gmail.com.
3. Alex van Duinen, Institute of Clinical and Molecular Medicine, Norwegian University of Science and Technology and Department of Surgery, St Olav's Hospital, Trondheim University Hospital, Trondheim, Norway. aalke.j.v.duinen@ntnu.no.
4. Anita Gadgil, Department of Surgery, BARC Hospital, WHO Collaboration Centre for Research in Surgical Care Delivery in LMICs, Mumbai, India, anitgadgil@gmail.com.
5. Juul Bakker, Médecins Sans Frontières, Amsterdam, Netherlands and Institute of Clinical and Molecular Medicine, Norwegian University of Science and Technology, juulbakker@gmail.com.
6. Pranav Bhushan, Senior Technical officer, Aspirational District Unit (ADU), Ministry of health and family welfare, GoI, New Delhi, Indiapranav.nru@gmail.com.

For peer review only - http://bmjopen.bmj.com/site/about/guidelines.xhtml

7. Nobhojit Roy, Department of Public Health Sciences, Karolinska Institute, Stockholm, Sweden nobhojit.roy@ki.se

¶These authors contributed equally to this work.

Keywords: Health Equity, Caesarean Section, LMIC, Maternal Health Services, Socioeconomic Factors

Word count: 3618

Title: Assessing geographical and economic inequalities in caesarean section rates between the districts of Bihar, India: A secondary analysis of the National Family Health Survey.

Abstract

Background: In Bihar, one of the most populous and poorest states in India, caesarean sections have increased over the last decade. However, an aggregated caesarean section rate at the state level may conceal inequities at the district level.

Objectives: The primary aim of this study was to analyse the inequalities in the geographic and socioeconomic distribution of caesarean sections between the districts of Bihar. The secondary aim was to compare the contribution of free-for-service government-funded public facilities and fee-for-service private facilities to the caesarean section rate.

Setting: Bihar, with a population in the 2011 census of approximately 104 million people, has a low GDP per capita (US$ 610), compared to other Indian states. The state has the highest crude birth rate (26.1 per 1,000 population) in India, with one baby born every two seconds. Bihar is divided into 38 administrative districts, 101 subdivisions and 534 blocks. Each district has a district (Sadar) hospital, and six districts also have one or more medical college hospitals.

For peer review only - http://bmjopen.bmj.com/site/about/guidelines.xhtml

Methods: This retrospective secondary data analysis was based on open-source national datasets from the 2015 and 2019 National Family Health Surveys, with respective sample sizes of 45,812 and 42,843 women aged 15–49 years.

Participants: Secondary data analysis of pregnant women delivering in public and private institutions.

Results: The caesarean section rate increased from 6.2% in 2015 to 9.7% in 2019 in Bihar. Districts with a lower proportion of poor population had higher caesarean section rates (R^2 = 0.45) among all institutional births, with 10.3% in private and 2.9% in public facilities. Access to private caesarean sections decreased (R^2 = 0.46) for districts with poorer populations.

Conclusion: Marked inequalities exist in access to caesarean sections. The public sector needs to be strengthened to improve access to obstetric services for those who need it most.

Article summary

- The strength of this study is its use of secondary data from India's largest and most reliable household-level survey data source, the National Family Health Survey (NFHS).
- A limitation of the study is that the NFHS-4 was used to determine wealth quintile data because the NFHS-5 had not yet released its wealth quintile data at the time of writing this paper.
- Evidence is mixed on the optimum caesarean section rates in the population for the maximum impact on reducing maternal mortality. The disaggregated cut-offs of 10% and 20% for adequate caesarean section rates in the population were considered practical.

For peer review only - http://bmjopen.bmj.com/site/about/guidelines.xhtml

Introduction

In many low- and middle-income countries (LMICs), poor access to life-saving caesarean sections is a risk factor for maternal death. The caesarean section rate serves as a proxy measure of the availability of comprehensive emergency obstetric and newborn care (CEmONC) services. A rate below 5% usually denotes a lack of access to CEmONC services. Though the 'optimum' caesarean section rate remains elusive, rates >20% are not associated with any further reduction in maternal deaths. (1–3)

In India, the national caesarean section rate is climbing and almost at par with global figures. Further, the maternal mortality ratio in India has decreased from 437 (1992–1993) to 113 (2016–2017), in a quarter of a century. (4,5) Government programs in India have contributed to increasing institutional deliveries, but the private sector has a strong role in increasing caesarean section rates. In addition, government maternal health programs, strong advocacy, and improved infrastructure and staffing have driven the increase in caesarean section rates in LMICs. (6)

Bihar, a relatively poor state in East India bordering Nepal, has seen an increase in the caesarean section rate from 6.2% in 2015 to 9.7% in 2019. (7,8) As this figure approaches 10%, it may suggest that the population's need for caesarean sections is met. Given the prevalent social inequalities, an optimum aggregate caesarean section rate at the state level may conceal inequities at the district level. Aggregated statistics conceal women who need but do not receive a caesarean section due to access or financial barriers by compensating with those who receive a caesarean section not medically indicated. Therefore, a more granular analysis of caesarean section rates at the district level is needed. (9)

For peer review only - http://bmjopen.bmj.com/site/about/guidelines.xhtml

In this paper, we report differences in caesarean section rates between population subgroups based on socioeconomic status across district wealth quintiles in Bihar. The study aimed to assess the geographical and economic inequalities in caesarean section rates between the districts of Bihar. In addition, the contributions of free government-funded public facilities and fee-for-service private facilities to population caesarean section rates are compared.

Methods

We conducted a secondary analysis of publicly available data from the National Family Health Survey (NFHS), a large-scale multi-round survey conducted in a representative sample of households throughout India. (7,10) The NFHS provides information on health and nutrition indicators at the sub-national level. The Government of India Ministry of Health and Family Welfare selected the International Institute for Population Sciences, Mumbai, as the nodal agency for the various rounds. The Woman's Schedule covered a wide variety of topics, including women's characteristics, marriage, fertility, contraception, children's immunisations and healthcare, nutrition, reproductive health, sexual behaviour, HIV/AIDS, women's empowerment and domestic violence, but not maternal deaths.

Data sources

For Bihar, the NFHS-5 data-collection was done in 2019–2020 from 35,834 households, which included 42,843 women aged 15–49 years. (7) The NFHS records whether a caesarean section was performed in a public or private facility. The wealth quintile data were available in the 2015–2016 NFHS-4 (8,10), and raw data for Bihar were provided by the nodal agency on request.

For peer review only - http://bmjopen.bmj.com/site/about/guidelines.xhtml

Study setting

Bihar, with a population in the 2011 census of approximately 104 million people (11), has a low GDP per capita (US$ 610) compared to other Indian states. (9,12) In total, 88% of the population of Bihar lives in rural areas. The state has the highest crude birth rate (26.1 per 1,000 population) in India, with one baby born every two seconds. (7) Bihar is divided into 38 administrative districts, 101 subdivisions and 534 blocks. Each district has a district (Sadar) hospital, and six districts also have one or more medical college hospitals.

Bihar's government healthcare system functions on three levels. At the primary level, sub-health centres and primary health centres function as the first point of contact. These primary facilities, providing basic emergency obstetric and newborn care (BEmONC) services, are mostly staffed by nurses, general physicians and alternative medicine practitioners but have no surgical capacity. At the secondary level, 149 first referral units (FRUs) function as first-level CEmONC units where caesarean sections are performed. These FRUs include district and sub-district hospitals (serving a population of 500,000–600,000) and community health centres (block-level hospitals), as per Indian Public Health Standards. (13) In addition, seven government and two autonomous medical colleges in Bihar are designated as tertiary centres, four of which are in the capital city of Patna (two government medical colleges and two autonomous medical colleges). Four medical colleges are located in North Bihar, four in Patna and only one in South Bihar. Medical colleges (though tertiary) fill a gap by also functioning as secondary health facilities because FRUs are poorly functional.

Of the mothers who delivered during the last five years in Bihar, 25.5% had at least four antenatal clinic visits and 89.5% were protected against neonatal tetanus. (7) Most

For peer review only - http://bmjopen.bmj.com/site/about/guidelines.xhtml

deliveries (76.2%) take place in an institution, with 56.9% in a public facility and 19.3% in a private facility.

According to the NFHS-4, (8) each household is assigned to a wealth quintile based on its household characteristics and scores established by the principal component analysis. (14). In Bihar, 80.2% of the population belongs to the two poorest quintiles, 11.4% to the middle quintile and only 8.36% to the two richest quintiles. (8) To compare districts, we combined the proportions of people in the two poorest quintiles.

Data analysis

Caesarean section rates and the proportion of institutional deliveries for each district were extracted from the NFHS-5, including the contributions by the public and private sectors. Since no optimum rate exists, we stratified the rates into <10%, 10–20% and >20% to measure equity in access to caesarean sections, from possible inadequate access to overuse. (15) The NFHS-4 and NFHS-5 caesarean section rates, respectively for 2015–2016 and 2019–2020, are presented in Figure 1 to demonstrate the geographical differences in change in rates between districts. Regression analysis was performed assessing the correlation between the proportion of the population in the two poorest quintiles and caesarean section rates. Both the caesarean section rate among all institutional births and the proportion of institutional deliveries in private and public health facilities were considered using the extrapolated caesarean section rate from the NFHS-5. (7)

For peer review only - http://bmjopen.bmj.com/site/about/guidelines.xhtml

Patient and public involvement

This is an analysis of secondary data in the public domain, collected in NFHS-4 and NFHS-5 in India. We have not performed direct patient recruitment or any intervention on the participants because individual patient data was not used and the reports of these surveys and various open-source data were analysed. The ethical approval for the NFHS-4 and NFHS-5 was obtained from the ethics review board of the International Institute for Population Sciences, Mumbai, India. These surveys were also reviewed and approved by the ICF International Review Board. Informed written consent for participation in this survey was obtained from the respondents during the survey. Each individual's approval was sought before the interview was conducted.

Results

Caesarean section rate trends across districts in Bihar

In Bihar, the caesarean section rate has increased from 6.2% in 2015 (NFHS-4) to 9.7% in 2019 (NFHS-5). Of the rate in 2019, 2.0% came from the public sector and 7.6% from the private sector. The NFHS-5 documented that the percentages of all live births delivered by caesarean section in public and private facilities were 3.6% and 39.6%, respectively.

Figure 1 shows the district-wise change in the rate of caesarean sections from 2015 to 2019. The number of districts with caesarean section rates below 10% decreased from 34 in 2015 to 21 in 2019. Using the 10% rate to evaluate access to caesarean sections in the 38 district populations, 14 districts were above 10% in 2019, three were at 10% and 21 were below 10%. Three districts (Jehanabad, Munger and Patna) had caesarean section rates above 20%.

For peer review only - http://bmjopen.bmj.com/site/about/guidelines.xhtml

The degree of change in caesarean section rates was uneven at the district level. Of the 38 districts, 28 demonstrated a remarkable increase of 5.9% in caesarean section rates in four years (from 5.5% in 2015 to 11.4% in 2019). However, against this trend was a 2.1% reduction in caesarean section rates in 10 districts (from 8.5% in 2015 to 6.5% in 2019).

Surrounding Patna are 10 adjacent districts within 86 kilometres or a two-hour driving time. In these districts, 77.4% of the population belongs to the two poorest wealth quintiles. Moving away from the capital city, we noted a decrease in the caesarean section rate to 11.3% (of which 9.3% were in private and 2.0% were in public facilities). The more remote districts are on average 192 kilometres away from the capital, or 5–6 hours transit. Of the population in these remote districts, 81.1% are in the two poorest quintiles, and the furthest from the capital had a further caesarean section rate drop to 9.7% (of which 7.5% were in private and 2.2% were in public facilities). Public facilities conducted proportionally more caesarean sections in districts with a larger proportion of people from poorer quintiles.

Figure 1: Caesarean section rate by district in 2015 and 2019

Comparison of caesarean section rate in the districts of Bihar in 2015 (NFHS-4) and 2019 (NFHS-5).

Among all institutional births at public facilities, the caesarean section rate markedly decreased from 8.2% in 2015 to 2.1% in 2019. Contrary to the overall rising trend of caesarean section rate, the number of government public facilities performing at least 10% caesarean sections among all deliveries dropped from eight in 2015 to zero in 2019. The districts with university teaching hospitals and medical colleges (Bhagalpur,

For peer review only - http://bmjopen.bmj.com/site/about/guidelines.xhtml

Nalanda, Darbhanga, Gaya, Muzaffarpur and Patna) in addition to district hospitals had district-level caesarean section rates higher than 10%, except West Champaran (6.4%).

In contrast, the caesarean section rate increased in private facilities from 6.8% in 2015 to 8.0% in 2019. Only four districts in 2015 had a caesarean section rate of >10% in private facilities, which increased to nine districts in 2019.

Caesarean section rate trends by district wealth quintiles in Bihar

Districts with higher proportions of the two richest quintiles (Bhagalpur, Buxar, Nalanda, Patna and Rohtas) had the highest caesarean section rates among all institutional births, with 11.8% in private facilities and 2.7% in public health facilities. Patna, the capital district of Bihar (and with the wealthiest people in the state), had the highest caesarean section rate in private facilities (17.9%) and a higher than average rate in public health facilities (4.0%). Supaul is the poorest district in Bihar, with 94.1% of the population in the lowest two quintiles; of all deliveries, 1.8% and 1.9% were delivered by caesarean respectively in private and public facilities.

Districts with a smaller proportion of population in the richest quintiles had lower caesarean section rates (Figure 2). The negative correlation (R^2 = 0.46) between population wealth quintiles and caesarean section rates in private health facilities demonstrates that access to private caesarean sections dropped dramatically in poorer populations. However, caesarean section rates dropped in poorer populations even in public facilities, though the correlation was weak (R^2 = 0.10). Public health facilities function independent of the wealth status of the population and are proportionately more available for the poor, who have lesser access to private care.

For peer review only - http://bmjopen.bmj.com/site/about/guidelines.xhtml

Figure 2: Proportion of total deliveries by caesarean section in private and public sector by wealth status in the districts of Bihar

The x-axis represents the proportion of the population classified in the two poorest wealth quintiles. The y-axis represents the proportion of all deliveries in the district delivered by caesarean section. For each district in Bihar, the contribution of the private sector (blue circle) and the public sector (orange square), together with the total rate of caesarean sections, are shown with individual markers. For each category, a trendline and the R^2 result are provided.

The institutional delivery rate in Bihar increased from 63.8% in 2005 to 76.2% in 2019. Overall institutional births showed a negative linear correlation (R^2 = 0.43) with the wealth status of the population, and homebirths were still associated with poorer populations (Figure 3). Populations in poorer districts are more reliant on public facilities for institutional deliveries and birth preparedness. With increasing poverty, institutional deliveries in private facilities decreased, with a negative linear correlation (R^2 = 0.64). Among the poorest two quintiles, the proportion of institutional deliveries in public facilities was higher than in private facilities. In the same group, the opposite was observed for caesarean sections, which are predominantly performed in the private sector.

Figure 3: Proportion of total deliveries in private and public institutions by wealth status in the districts of Bihar.

The x-axis represents the proportion of the population classified in the two poorest wealth quintiles. The y-axis represents the proportion of all deliveries in the district born in an institution. For each district in Bihar, the contribution of the private sector (blue

For peer review only - http://bmjopen.bmj.com/site/about/guidelines.xhtml

circle) and the public sector (orange square), together with the total institutional delivery rate are shown with individual markers. For each category, a trendline and the R^2 result are provided.

Discussion

In Bihar, the caesarean section rate increased from 6.2% in 2015 to 9.7% in 2019. However, caesarean sections were not equally distributed, and districts with a larger proportion of poor people had a lower rate of institutional deliveries and a lower rate of caesarean section rates compared to districts with a higher proportion of people in the two richest quintiles. The contribution of the private sector to the overall caesarean section rate was high, but it was lower in the poorer districts, while the contribution of the public sector was approximately the same.

Caesarean sections and the health system

Caesarean section rates are driven by supply-side (such as resources within the health system, healthcare policy and strategies, health financing systems and perceptions of healthcare professionals) and demand-side (such as socioeconomic status, population preference and perceptions and trust in the health system) determinants. This study reinforces the importance of government maternal health programs in promoting institutional deliveries in resource-poor economies. The poor population chooses to deliver in public health facilities because they are affordable and incentivised. In the poorest regions, establishing private facilities is not profitable because the paying capacity of the population is low, and therefore the private option is simply not available. The scale-up of institutional deliveries has been possible due to extensive and prolonged

For peer review only - http://bmjopen.bmj.com/site/about/guidelines.xhtml

training programs for auxiliary nurses and general nurse midwives, supported by task-shifted cadres of helpers in the labour delivery suite. (16)

Uneven caesarean section rates across geographical distribution

Bihar is divided into North and South Bihar, with marked variations in geography and health. (17) North Bihar is downstream of the seasonally flooding Himalayan rivers, whereas South Bihar is in the drought zone. In both areas, the availability and utilisation of health service facilities are driven by the socioeconomic status of the population and provider factors rather than physical access. (16) Geographical access to health facilities has improved in Bihar, with better roads, electricity and transportation over the past decade. (10) The eastern districts in Bihar are insecure workplaces for healthcare professionals due to political instability. Bihar has the lowest population-to-doctor ratio in the country, and the available doctors tend to choose urban areas for government service or private practice. (18)

The caesarean section rate varies across the districts of Bihar. We found that districts closer to the capital city of Patna had higher caesarean section rates. Other factors influencing the caesarean section rate were the number of active specialists, availability of private facilities and blood availability. All these factors decrease with distance from the capital. The caesarean section rates in Bihar display the entire spectrum from too little (inadequate access) to too much (overuse), all within one state. The powerful global acceptance of technological solutions and medicalising birth is set against the local traditional preference for vaginal delivery, regardless of educational status and across wealth quintiles in the Bihar population. The poorer population faces affordability and access barriers to caesarean sections. (18) Cultural beliefs and practices about vaginal birth, irrespective of wealth quintiles, make it the preferred mode of delivery over

For peer review only - http://bmjopen.bmj.com/site/about/guidelines.xhtml

caesarean section. Performing a caesarean section is still viewed as a lack of competence of the provider to perform an unassisted or assisted vaginal delivery, but this may also simply reflect a lack of trust or fear of a surgical procedure. Research has shown that demand for caesarean sections has been relatively uncommon in Bihar (16,18–21) due to cosmetic concerns, fear of pain during childbirth and sexual functioning concerns, foregrounded by the upper wealth quintiles from other nations (2) or other Indian states (19).

<u>Distribution of caesarean section rates between public and private health facilities</u>

Free-service public facilities are the first point of access for women with high-risk pregnancies in this predominantly poor state. However, while BEmONC services have been strengthened in public facilities, our findings show a quantitative reduction in CEmONC services available to the same women who chose to deliver in public facilities. While more than six out of 10 women delivered in public facilities, only around one in 28 women (3.6%) received a caesarean section in these public hospitals. Two in five children were delivered by caesarean section in private hospitals (39.6%). A proportionate decline occurred in the outputs and performance of public health facilities in Bihar over five years (2015–2019). Of 149 designated public FRUs, only 40 provide some CEmONC services, usually during office hours. The better-functioning public facilities, with significantly higher caesarean section rates, are attributed to the presence of medical colleges, teams of specialist doctors and greater urbanisation in those districts. (2) In the poorer-performing districts, low output is commonly attributed to inadequate infrastructure and staffing. However, rates remain low even in well-resourced public facilities with adequate infrastructure. (22) Our results show that caesarean section services in districts with the largest proportion of population in the poorest wealth quintiles have poor access to care in both public and private health facilities.

For peer review only - http://bmjopen.bmj.com/site/about/guidelines.xhtml

The supply-side provider barriers in public facilities are hesitancy to treat at-risk pregnant women, lack of clinical support for complications, administrative support for litigation, and occasional episodes of violence against healthcare workers. Government doctors who refuse to perform caesarean sections in public facilities are willing to operate on the same high-risk women in private facilities. Professional social networks provide a safety net and allow for higher risk-taking in private facilities, though the infrastructure is better in public facilities. Financial incentives to providers via government schemes (Janani Shishu Suraksha Karyakram) with performance-based incentives for doctors have not increased the provision of caesarean sections at government facilities. (17) Women with high-risk pregnancies are referred to higher-level facilities (private facilities, medical colleges or tertiary facilities) for caesarean sections. (23) The demand-side patient barriers for low uptake of caesarean sections include user fees, sociocultural beliefs around the value of the procedure, a longer stay in the hospital and a trust deficit about the safety of caesarean sections. Costs in private hospitals are at least two to four times those in public hospitals. (7,24) The fee structure in the private sector is not regulated by the government, and the private sector is therefore free to decide user fees. Currently, the predominantly urban private sector in Bihar is still inadequate to provide for the needs of the 80% of the population in the two poorest wealth quintiles. Therefore public facilities need to be functional to provide free and affordable caesarean section services.

Caesarean section distribution across wealth quintiles and equitable solutions

Caesarean section is a life-saving operation that should be available to all women. The probable reasons for the inequity and unaffordability of healthcare services include insufficient public financing and consequent out-of-pocket expenditure to meet the high costs of caesarean section services. (25)

For peer review only - http://bmjopen.bmj.com/site/about/guidelines.xhtml

To address inequity in access, the Government of India launched a conditional cash-transfer scheme to promote institutional deliveries by providing free services to neonates and pregnant women, aiming to encourage marginalised pregnant women to use free public health facilities. More recently, the world's largest health insurance scheme, Pradhan Mantri Jan Arogya Yojana (PM-JAY), was launched in 2018 to provide free services for the 500 million poor in India. Caesarean section is included in the PM-JAY maternal package to mitigate the impact of the catastrophic expenditure incurred. However, the overall uptake of PM-JAY reimbursement rates by private providers remains poor in the state. (26)

Lessons can be learned from other LMICs, such as Cambodia, Indonesia and Ghana, which have previously introduced policies to remove user fees for pregnant women. (2) Rwanda has set an example with its community-based health insurance scheme to reduce out-of-pocket payments for patients and a performance-based financing system to incentivise providers. (27) The key findings from our results – that although the overall caesarean rate has increased among the poor, the rates for the poorest subgroup still fall short – are also reflected by findings from Ghana and Rwanda. Like Ghana, Bihar exempts women from paying for delivery care and has a state policy of demand-side financing by incentivising institutional deliveries to reduce barriers to access to quality care in remote areas. Cambodia has identified and targeted the poor for user fee exemptions and health insurance provision through health equity funds. This scheme is similar to Indonesia's social health insurance program, *Jamkesmas*, for poor and near-poor people, which has been shown to improve access and reduce financial barriers. (28) In Bhutan, only small differences in caesarean section rates between wealth quintiles have been observed, with around one in eight of all deliveries by caesarean section. However, no privatisation of healthcare exists in this LMIC. (2,29) Like Bihar, Burundi

For peer review only - http://bmjopen.bmj.com/site/about/guidelines.xhtml

demonstrates increasing inequalities and caesarean section disparities with improved access to maternal healthcare. (30)

Strengths and limitations

A strength of this study is its use of secondary data from India's largest and most reliable household-level survey data sources, the NFHS. It also provides insight on a more granular level into the distribution of caesarean sections on district level and its relation to economic status. Finally, the role of the public sector is an important element that was considered and essential for the understanding of the distribution of caesarean sections.

A limitation of this study is that economic status was retrieved from the NFHS-4 because the NFHS-5 had not yet released its wealth quintile data at the time of writing. In addition, no clear consensus exists on an exact optimum caesarean section rate for maximum impact on reducing maternal mortality and minimal risk for unnecessary interventions. Therefore, the cut-offs of 10% and 20% were pragmatically chosen based on the available evidence.

Conclusion

Aggregated state-level caesarean section rates conceal the inequity of adequate access to emergency obstetric care among the poorest population, which is compensated by the high levels of caesarean use in the richest and most advantaged subgroups. Using relevant equity stratifiers, population subgroups and specific strategies need to be identified to address the inequities by improving access to maternal and newborn health services in public health facilities. The role of the private sector in meeting the demand for caesarean sections for the population is strongly substantiated, but the concomitant out-of-pocket expenditure is debilitating and catastrophic.

For peer review only - http://bmjopen.bmj.com/site/about/guidelines.xhtml

Contributorship statement: Nobhojit Roy, Anita Gadgil and Alex van Duinen conceptualised and planned the research project. Minal Shukla, Monali Mohan, Juul Bakker and Pranav Bhushan conducted the analysis. All contributed to the writing of the manuscript.

Acknowledgments: We thank Priti Patil for her input in the manuscript.

Competing interests: None of the authors have any competing interests.

Funding: It was a non-funded project.

Data sharing statement: Data are available in a public, open access repository, additionally, data can be shared by the corresponding author.

Ethics approval statement: This was an analysis of secondary data from open access and no primary data has been collected from human subjects.

References

1. Sobhy S, Arroyo-Manzano D, Murugesu N, Karthikeyan G, Kumar V, Kaur I, et al. Maternal and perinatal mortality and complications associated with caesarean section in low-income and middle-income countries: a systematic review and meta-analysis. Lancet [Internet]. 2019;393(10184):1973–82. Available from: http://dx.doi.org/10.1016/S0140-6736(18)32386-9

2. Boatin AA, Schlotheuber A, Betran AP, Moller AB, Barros AJD, Boerma T, et al. Within country inequalities in caesarean section rates: Observational study of 72 low and middle income countries. BMJ. 2018;360.

3. Molina G, Weiser TG, Lipsitz SR, Esquivel MM, Uribe-Leitz T, Azad T, et al. Relationship between cesarean delivery rate and maternal and neonatal mortality. JAMA - J Am Med Assoc. 2015;314(21):2263–70.

For peer review only - http://bmjopen.bmj.com/site/about/guidelines.xhtml

4. International Institute for Population Sciences. National Family Health Survey (NFHS-1) 1992-1993 India. Int Inst Popul Sci [Internet]. 1995. Available from: http://rchiips.org/nfhs/india1.shtml

5. Sample Registration System: Special Bulletin on Maternal Mortality in India 2016-2018 [Internet]. New Delhi; 2020. Available from: https://censusindia.gov.in/vital_statistics/SRS_Bulletins/MMR%20Bulletin%202016-18.pdf

6. Meara JG, Leather AJM, Hagander L, Alkire BC, Alonso N, Ameh EA, et al. Global Surgery 2030: evidence and solutions for achieving health, welfare, and economic development. Lancet [Internet]. 2015;386(9993):569–624. Available from: http://www.ncbi.nlm.nih.gov/pubmed/25924834?otool=karolib&tool=karolinska

7. International Institute for Population Sciences. National Family Health Survey-5 (Bihar State Report) 2019-2020 [Internet]. Mumbai; 2020. Available from: http://rchiips.org/nfhs/NFHS-5_FCTS/FactSheet_BR.pdf

8. International Institute for Population Sciences. National Family Health Survey (NFHS-4) 2015-16 India. Int Inst Popul Sci ICF [Internet]. 2017;1–192. Available from: http://rchiips.org/NFHS/NFHS-4Reports/India.pdf

9. Guilmoto CZ, Dumont A. Trends, Regional Variations, and Socioeconomic Disparities in Cesarean Births in India, 2010-2016. JAMA Netw open. 2019;2(3):e190526.

10. International Institute for Population Sciences. National Family Health Survey-4 (Bihar State Report) 2015-2016 [Internet]. Mumbai; 2020. Available from: http://rchiips.org/nfhs/NFHS-4Reports/Bihar.pdf

11. Census Organization of India. Census of India 2011: Bihar [Internet]. [cited 2020 Jul

For peer review only

20]. Available from: http://www.census2011.co.in/census/state/bihar.html

12. Srivastava S, Chaurasia H, Kumar Singh KJ, Chaudhary P. Exploring the spatial patterns of cesarean section delivery in India: Evidence from National Family Health Survey-4. Clin Epidemiol Glob Heal [Internet]. 2019;(September):1–9. Available from: https://doi.org/10.1016/j.cegh.2019.09.012

13. Directorate General of Health Services Ministry of Health & Family Welfare Government of India. Indian Public Health Standards (IPHS) Guidelines for Community Health Centres [Internet]. New Delhi; 2012. Available from: http://nhm.gov.in/images/pdf/guidelines/iphs/iphs-revised-guidlines-2012/community-health-centres.pdf

14. Rutstein SO. Steps to constructing the new DHS Wealth Index [Internet]. 2014. Available from: https://dhsprogram.com/programming/wealth index/Steps_to_constructing_the_new_DHS_Wealth_Index.pdf

15. Betran AP, Torloni MR, Zhang JJ, Gülmezoglu AM. WHO statement on caesarean section rates. BJOG An Int J Obstet Gynaecol. 2016;123(5):667–70.

16. Creanga AA, Srikantiah S, Mahapatra T, Das A, Sonthalia S, Ranjan Moharana P, et al. Statewide implementation of a quality improvement initiative for reproductive, maternal, newborn and child health and nutrition in Bihar, India. J Glob Health. 2020;10(2):1–13.

17. Salazar M, Vora K, Annerstedt KS, De Costa A. Caesarean sections in the in the context of the Chiranjeevi Yojana public private partnership program to promote institutional birth in Gujarat, India; Does the embedded disincentive for caesarean section work? Int J Equity Health. 2019;18(1):1–8.

For peer review only - http://bmjopen.bmj.com/site/about/guidelines.xhtml

18. Kaur J, Franzen SRP, Newton-Lewis T, Murphy G. Readiness of public health facilities to provide quality maternal and newborn care across the state of Bihar, India: A cross-sectional study of district hospitals and primary health centres. BMJ Open. 2019;9(7):1–10.

19. Vora KS, Yasobant S, Sengupta R, De Costa A, Upadhyay A, Mavalankar D V. Options for optimal coverage of free C section services for poor mothers in indian state of Gujarat: Location allocation analysis using GIS. PLoS One. 2015;10(9):1–16.

20. Office of the Registrar General & Census Commissioner. Annual Health Survey:2011-12 Fact Sheet Bihar. 2012;1–176. Available from: www.censusindia.gov.in

21. Dhirar N, Kumar R, Dudeja S. Upward trend of caesarean sections in India: Incision over precision. Vol. 31, National Medical Journal of India. 2018. p. 316–7.

22. National Health Mission. 12th Common Review Mission [Internet]. New Delhi; 2019 [cited 2021 May 2]. Available from: http://nhsrcindia.org/sites/default/files/12th Common Review Mission-Report 2018.pdf

23. Bhatia M, Banerjee K, Dixit P, Dwivedi LK. Assessment of Variation in Cesarean Delivery Rates Between Public and Private Health Facilities in India From 2005 to 2016. JAMA Netw open. 2020;3(8):e2015022.

24. Radhakrishnan A, Vasanthakumari K, PK B. Increasing Trend of Caesarean Rates in India: Evidence from NFHS-4. J Med Sci Clin Res [Internet]. 2017 Aug 7;5(8):26167–76. Available from: http://jmscr.igmpublication.org/v5-i8/31 jmscr.pdf

25. Balarajan Y, Selvaraj S, Subramanian S. Health care and equity in India. Lancet [Internet]. 2011 Feb;377(9764):505–15. Available from: https://linkinghub.elsevier.com/retrieve/pii/S0140673610618946

For peer review only - http://bmjopen.bmj.com/site/about/guidelines.xhtml

26. National Health Authority. Report on Top Utilized Private Hospitals - Ayushman Bharat- Pradhan Mantri Jan Arogya Yojana (AB PM-JAY) [Internet]. New Delhi; 2020. Available from: https://pmjay.gov.in/sites/default/files/2019-10/Report on top utilized pvt hospitals_Final.pdf

27. Gibbons L, Belizán J, Lauer J, Betrán A, Merialdi M, Althabe F. The Global Numbers and Costs of Additionally Needed and Unnecessary Caesarean Sections Performed per Year: Overuse as a Barrier to Universal Coverage HEALTH SYSTEMS FINANCING [Internet]. Vol. 30, World Health Report 2010. 2010 Jan.

28. Zahroh RI, Disney G, Betrán AP, Bohren MA. Trends and sociodemographic inequalities in the use of caesarean section in Indonesia, 1987-2017. BMJ Glob Heal. 2020;5(12).

29. Hanson C, Betrán AP, Opondo C, Mkumbo E, Manzi F, Mbaruku G, et al. Trends in caesarean section rates between 2007 and 2013 in obstetric risk groups inspired by the Robson classification: results from population-based surveys in a low-resource setting. BJOG An Int J Obstet Gynaecol. 2019;126(6):690–700.

30. Yaya S, Zegeye B, Idriss-Wheeler D, Shibre G. Inequalities in caesarean section in Burundi: Evidence from the Burundi Demographic and Health Surveys (2010-2016). BMC Health Serv Res. 2020;20(1):1–8.

For peer review only

For peer review only - http://bmjopen.bmj.com/site/about/guidelines.xhtml

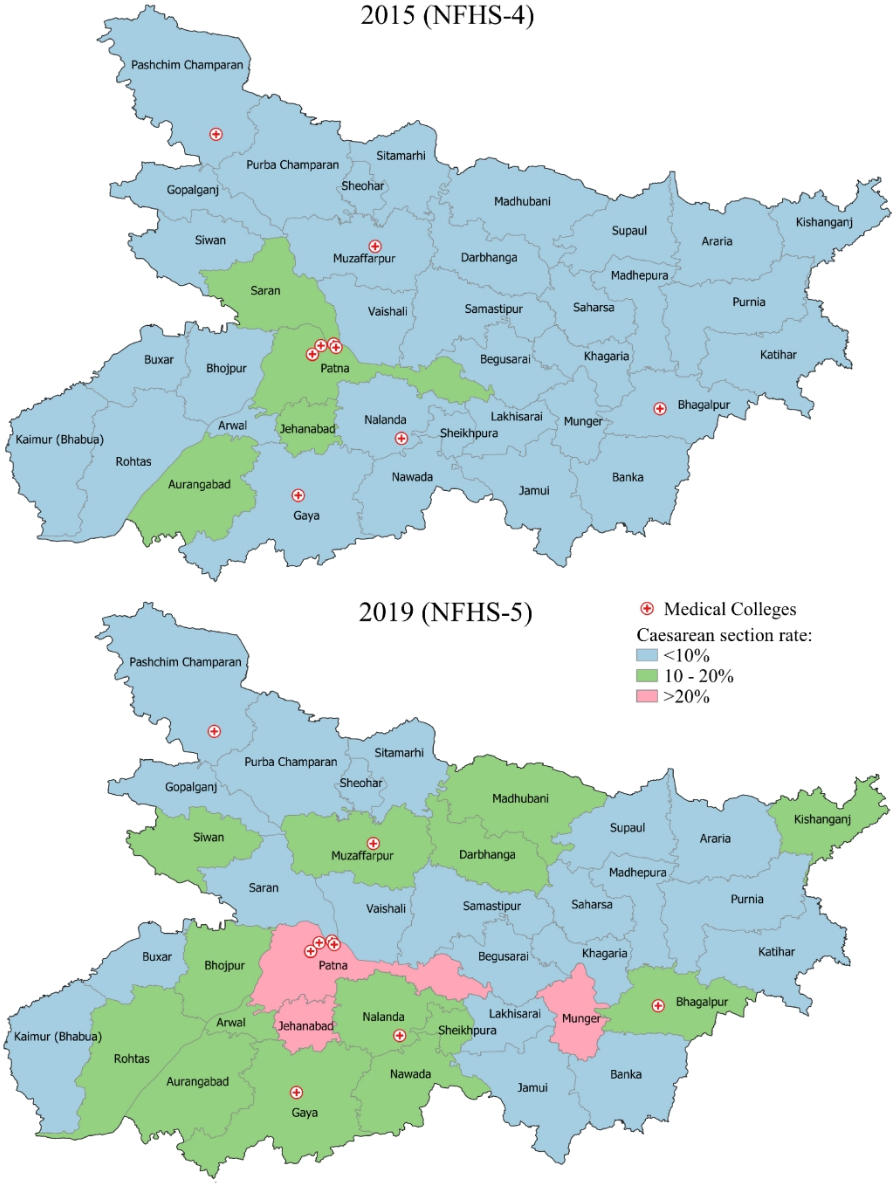

Comparison of caesarean section rate in the districts of Bihar in 2015 (NFHS-4) and 2019 (NFHS-5).

209x280mm (300 x 300 DPI)

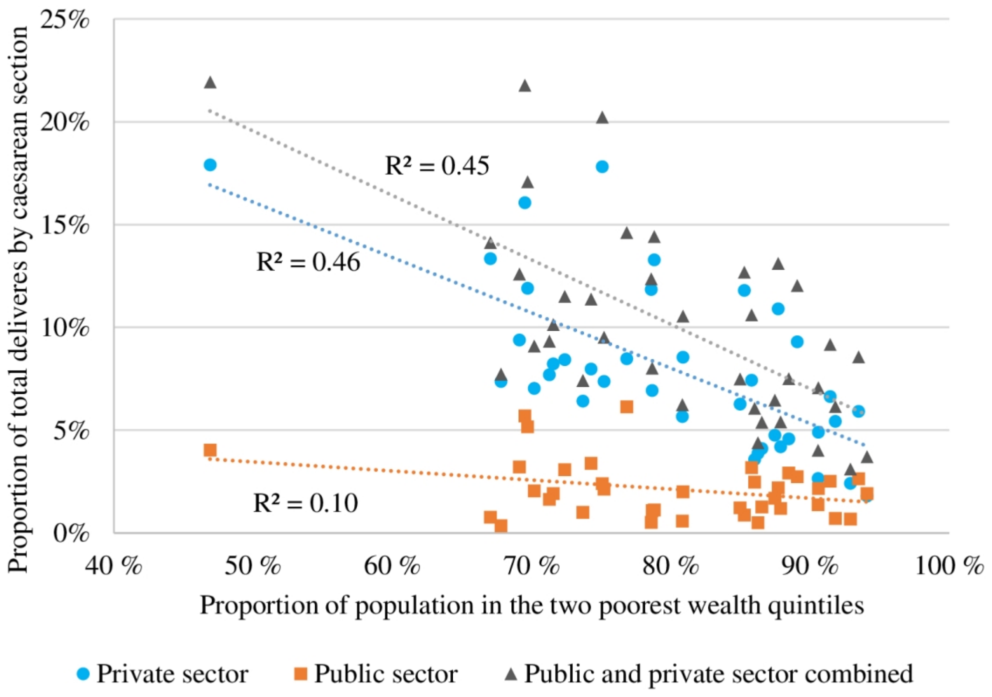

The x-axis represents the proportion of the population classified in the two poorest wealth quintiles. The y-axis represents the proportion of all deliveries in the district delivered by caesarean section. For each district in Bihar, the contribution of the private sector (blue circle) and the public sector (orange square), together with the total rate of caesarean sections, are shown with individual markers. For each category, a trendline and the R^2 result are provided.

132x93mm (300 x 300 DPI)

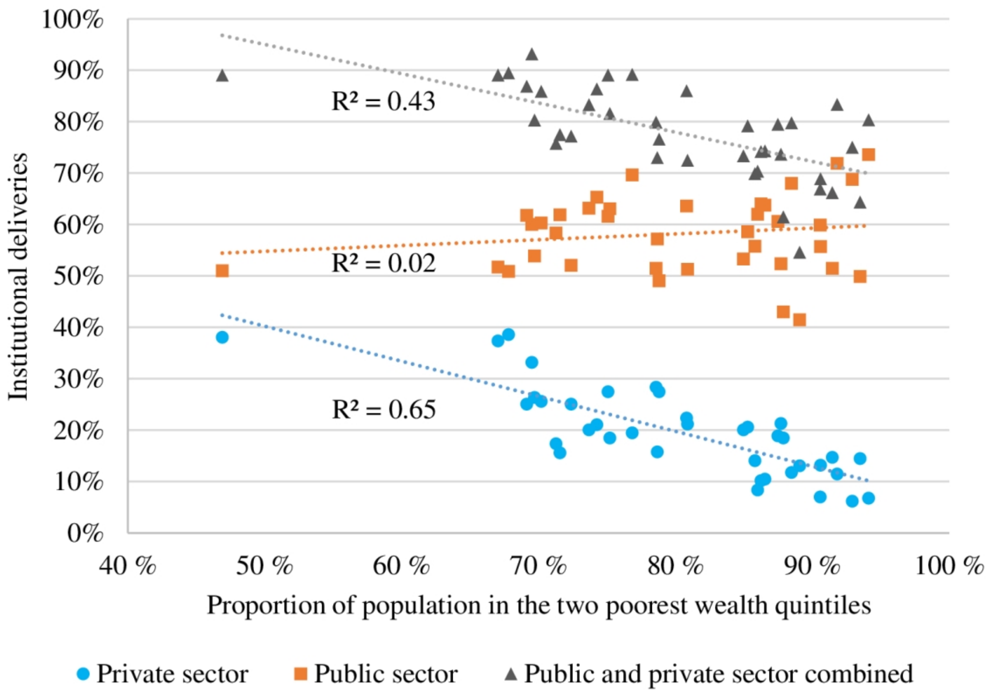

The x-axis represents the proportion of the population classified in the two poorest wealth quintiles. The y-axis represents the proportion of all deliveries in the district born in an institution. For each district in Bihar, the contribution of the private sector (blue circle) and the public sector (orange square), together with the total institutional delivery rate are shown with individual markers. For each category, a trendline and the R^2 result are provided.

132x93mm (300 x 300 DPI)

STROBE CHECKLIST			
**Section/topic**	**Item number**	**Page Number**	**Recommendation**
**Title and abstract**	1	1-3	**Title**: Assessing geographical and economic inequalities in caesarean section rates between the districts of Bihar, India: A secondary analysis of the National Family Health Survey. **Abstract:** In Bihar, one of the most populous and poorest states in India, caesarean sections have increased over the last decade. By retrospective secondary data analysis based on open-source national datasets from the 2015 and 2019 National Family Health Surveys we found there is marked inequalities exist in access to caesarean sections. The public sector needs to be strengthened to improve access to obstetric services for those who need it most.
**Introduction**	2	4	
Background/rationale	3	NA	In India caesarean section rates are now almost at par with the global figures. In Bihar, which is most populous and poorest states in India caesarean sections increased over the last decade. Though state level aggregate caesarean rates in Bihar have risen to be near global figures, it conceals the inequities at the district level and vary as per economic status of the communities.
Objectives	4	5	The study aimed to assess the geographical and economic inequalities in caesarean section rates between the districts of Bihar. In addition, the contributions of free government-funded public facilities and fee-for-

STROBE CHECKLIST			
**Section/topic**	**Item number**	**Page Number**	**Recommendation**
			service private facilities to population caesarean section rates are compared.
**Methods**		5	
Study design	5	5	It is a Cross sectional study of secondary data study based on open source national datasets from 'National Family Health Surveys' of last two rounds in India (NFHS-4 (2015-16) and NFHS-5 (2019-20).
Setting	6	6	Study setting is the public health facilities and private facilities of northern state of India Bihar. It is a secondary data analysis from National Family Health Survey.
Participants	7	5	Population covered in the two rounds of national survey from Bihar, India.
Variables	8	NA	Total caesarean section rates, caesarean section rates in Private facilities and C section rates in public facilities in the districts of Bihar as available from the National survey . Wealth quintile data of the population covered in Bihar

STROBE CHECKLIST			
**Section/topic**	**Item number**	**Page Number**	**Recommendation**
Data sources/measurement	9*	5	This retrospective secondary data analysis was based on open source national datasets from 'National Family Health Surveys' of last two rounds.
Bias	10	NA	The Government of India Ministry of Health and Family Welfare selected the International Institute for Population Sciences, Mumbai, as the nodal agency for the various rounds of this survey. Sample from Bihar gathered from 35,834 households, 42,483 women, and 4,897 men.
Study size	11	5	Entire data set collected from Bihar regarding caesarean section and distribution of population across wealth quintiles was used. No sample drawn from this
Quantitative variables	12	6	According to the NFHS-4 the Indian population is divided into wealth quintiles. Based on this division, the proportion in each of these wealth quintiles for each district has been published.
Statistical methods	13	7	Statistical methods used is regression analysis. Population sub-groups falling under two poorest wealth quintile pregnant women sought for delivery services in public and private health facilities.

STROBE CHECKLIST			
**Section/topic**	**Item number**	**Page Number**	**Recommendation**
Results	14	8	
Participants	15*	5	Entire data set collected from Bihar regarding caesarean section and distribution of population across wealth quintiles was used. No sample drawn from this
Descriptive data	16*	NA	Not applicable
Outcome data	17*	8	Distribution of C sections across various wealth quintiles at districts in Bihar. Distribution of caesarean sections done in private and public facilities across wealth quintiles
Main results	18	8-12	Caesarean section rate increased from 6.20% in 2015 to 9.70% in 2019 in Bihar. Districts with a lower proportion of poor had higher caesarean section rates (R2=0.45) among all institutional births with 10.32% among them in private and 2.89% in public facilities. Access to private caesarean sections decreased (R2=0.46) for districts with poorer populations.
Other analyses	19	NA	Not applicable.

STROBE CHECKLIST			
**Section/topic**	**Item number**	**Page Number**	**Recommendation**
Discussion	20	8-16	Across the LMIC inequality in health care access is evident because of various reasons like geography, economic conditions etc. Population sub group in poor wealth quintile agonize for access of services like institutional delivery and caesarean section in public as well as private health facilities. However government made many policy level decisions to reduce the hurdles for poor.
Key results	21	12	Caesarean section rate increased from 6.20% in 2015 to 9.70% in 2019 in Bihar. Districts with a lower proportion of poor had higher caesarean section rates (R2=0.45) among all institutional births with 10.32% among them in private and 2.89% in public facilities. Access to private caesarean sections decreased (R2=0.46) for districts with poorer populations.
Limitations	22	17	A limitation of this study is that economic status was retrieved from the NFHS-4 because the NFHS-5 had not yet released its wealth quintile data at the time of writing. In addition, no clear consensus exists on an exact optimum caesarean section rate for maximum impact on reducing maternal mortality and minimal risk for unnecessary interventions. Therefore, the cut-offs of 10% and 20% were pragmatically chosen based on the available evidence.

STROBE CHECKLIST			
**Section/topic**	**Item number**	**Page Number**	**Recommendation**
Interpretation	23	8-16	There are marked inequalities in access to caesarean sections. Public sector need to strengthen to maintain access to obstetric services for those that need it from poorer wealth quintiles
Generalizability	24	NA	Not applicable.
Other information	25		
Funding	26	18	Non- funded research.

w only

BMJ Open

Assessing geographical and economic inequalities in caesarean section rates between the districts of Bihar, India: A secondary analysis of the National Family Health Survey

Journal:	*BMJ Open*
Manuscript ID	bmjopen-2021-055326.R2
Article Type:	Original research
Date Submitted by the Author:	15-Oct-2021
Complete List of Authors:	Shukla, Minal; WHO Collaboration Centre for Research in Surgical Care Delivery in LMICs<br>Mohan, Monali; WHO Collaboration Centre for Research in Surgical Care Delivery in LMICs, Mumbai, India,<br>van Duinen, Alex; NTNU Fakultet for ingeniorvitenskap og teknologi Trondheim, Faculty of Medicine and Health Sciences Department of Cancer Research and Molecular Medicine; Sankt Olavs Hospital Universitetssykehuset i Trondheim, Department of Surgery<br>Gadgil, Anita; World Health Organization Collaborating Center for Research in Surgical Care Delivery in Low-and-Middle Income Countries<br>Bakker, Juul; Médecins Sans Frontières, Amsterdam, Netherlands<br>Bhushan, Pranav ; Ministry of health and family welfare, GoI<br>Roy, Nobhojit; Karolinska Institutet, Department of Global Public Health
**Primary Subject Heading**:	Health services research
Secondary Subject Heading:	Obstetrics and gynaecology, Surgery
Keywords:	OBSTETRICS, EPIDEMIOLOGY, Health policy < HEALTH SERVICES ADMINISTRATION & MANAGEMENT

SCHOLARONE™
Manuscripts

I, the Submitting Author has the right to grant and does grant on behalf of all authors of the Work (as defined in the below author licence), an exclusive licence and/or a non-exclusive licence for contributions from authors who are: i) UK Crown employees; ii) where BMJ has agreed a CC-BY licence shall apply, and/or iii) in accordance with the terms applicable for US Federal Government officers or employees acting as part of their official duties; on a worldwide, perpetual, irrevocable, royalty-free basis to BMJ Publishing Group Ltd ("BMJ") its licensees and where the relevant Journal is co-owned by BMJ to the co-owners of the Journal, to publish the Work in this journal and any other BMJ products and to exploit all rights, as set out in our licence.

The Submitting Author accepts and understands that any supply made under these terms is made by BMJ to the Submitting Author unless you are acting as an employee on behalf of your employer or a postgraduate student of an affiliated institution which is paying any applicable article publishing charge ("APC") for Open Access articles. Where the Submitting Author wishes to make the Work available on an Open Access basis (and intends to pay the relevant APC), the terms of reuse of such Open Access shall be governed by a Creative Commons licence – details of these licences and which Creative Commons licence will apply to this Work are set out in our licence referred to above.

Other than as permitted in any relevant BMJ Author's Self Archiving Policies, I confirm this Work has not been accepted for publication elsewhere, is not being considered for publication elsewhere and does not duplicate material already published. I confirm all authors consent to publication of this Work and authorise the granting of this licence.

For peer review only

Title page:

Title: Assessing geographical and economic inequalities in caesarean section rates between the districts of Bihar, India: A secondary analysis of the National Family Health Survey.

Corresponding author.

Dr Nobhojit Roy: Address: 601, Balaji Apartment, Nerul sec 15, Navi Mumbai, Maharashtra India. Email: nobhojit.roy@ki.se; Mobile Number: +91 98212 91225

Authors

1. Minal Shukla¶, Global Public Health Expert WHO Collaboration Centre for Research in Surgical Care Delivery in LMICs, Mumbai, India, shukla.minal@gmail.com.
2. Monali Mohan¶, Consultant, WHO Collaboration Centre for Research in Surgical Care Delivery in LMICs, Mumbai, India, monalimohangupta@gmail.com.
3. Alex van Duinen, Institute of Clinical and Molecular Medicine, Norwegian University of Science and Technology and Department of Surgery, St Olav's Hospital, Trondheim University Hospital, Trondheim, Norway. aalke.j.v.duinen@ntnu.no.
4. Anita Gadgil, Department of Surgery, BARC Hospital, WHO Collaboration Centre for Research in Surgical Care Delivery in LMICs, Mumbai, India, anitgadgil@gmail.com.
5. Juul Bakker, Médecins Sans Frontières, Amsterdam, Netherlands and Institute of Clinical and Molecular Medicine, Norwegian University of Science and Technology, juulbakker@gmail.com.
6. Pranav Bhushan, Senior Technical officer, Aspirational District Unit (ADU), Ministry of health and family welfare, GoI, New Delhi, Indiapranav.nru@gmail.com.

For peer review only - http://bmjopen.bmj.com/site/about/guidelines.xhtml

7. Nobhojit Roy, Department of Public Health Sciences, Karolinska Institute, Stockholm, Sweden nobhojit.roy@ki.se

¶These authors contributed equally to this work.

Keywords: Health Equity, Caesarean Section, LMIC, Maternal Health Services, Socioeconomic Factors

Word count: 3618

Title: Assessing geographical and economic inequalities in caesarean section rates between the districts of Bihar, India: A secondary analysis of the National Family Health Survey.

Abstract

Background: In Bihar, one of the most populous and poorest states in India, caesarean sections have increased over the last decade. However, an aggregated caesarean section rate at the state level may conceal inequities at the district level.

Objectives: The primary aim of this study was to analyse the inequalities in the geographic and socioeconomic distribution of caesarean sections between the districts of Bihar. The secondary aim was to compare the contribution of free-for-service government-funded public facilities and fee-for-service private facilities to the caesarean section rate.

Setting: Bihar, with a population in the 2011 census of approximately 104 million people, has a low GDP per capita (US$ 610), compared to other Indian states. The state has the highest crude birth rate (26.1 per 1,000 population) in India, with one baby born every two seconds. Bihar is divided into 38 administrative districts, 101 subdivisions and 534 blocks. Each district has a district (Sadar) hospital, and six districts also have one or more medical college hospitals.

For peer review only - http://bmjopen.bmj.com/site/about/guidelines.xhtml

Methods: This retrospective secondary data analysis was based on open-source national datasets from the 2015 and 2019 National Family Health Surveys, with respective sample sizes of 45,812 and 42,843 women aged 15–49 years.

Participants: Secondary data analysis of pregnant women delivering in public and private institutions.

Results: The caesarean section rate increased from 6.2% in 2015 to 9.7% in 2019 in Bihar. Districts with a lower proportion of poor population had higher caesarean section rates (R^2 = 0.45) among all institutional births, with 10.3% in private and 2.9% in public facilities. Access to private caesarean sections decreased (R^2 = 0.46) for districts with poorer populations.

Conclusion: Marked inequalities exist in access to caesarean sections. The public sector needs to be strengthened to improve access to obstetric services for those who need it most.

Article summary

- The strength of this study is its use of secondary data from India's largest and most reliable household-level survey data source, the National Family Health Survey (NFHS).
- A limitation of the study is that the NFHS-4 was used to determine wealth quintile data because the NFHS-5 had not yet released its wealth quintile data at the time of writing this paper.
- Evidence is mixed on the optimum caesarean section rates in the population for the maximum impact on reducing maternal mortality. The disaggregated cut-offs of 10% and 20% for adequate caesarean section rates in the population were considered practical.

For peer review only - http://bmjopen.bmj.com/site/about/guidelines.xhtml

Introduction

In many low- and middle-income countries (LMICs), poor access to life-saving caesarean sections is a risk factor for maternal death. The caesarean section rate serves as a proxy measure of the availability of comprehensive emergency obstetric and newborn care (CEmONC) services. A rate below 5% usually denotes a lack of access to CEmONC services. Though the 'optimum' caesarean section rate remains elusive, rates >20% are not associated with any further reduction in maternal deaths. (1–3)

In India, the national caesarean section rate is climbing and almost at par with global figures. Further, the maternal mortality ratio in India has decreased from 437 (1992–1993) to 113 (2016–2017), in a quarter of a century. (4,5) Government programs in India have contributed to increasing institutional deliveries, but the private sector has a strong role in increasing caesarean section rates. In addition, government maternal health programs, strong advocacy, and improved infrastructure and staffing have driven the increase in caesarean section rates in LMICs. (6)

Bihar, a relatively poor state in East India bordering Nepal, has seen an increase in the caesarean section rate from 6.2% in 2015 to 9.7% in 2019. (7,8) As this figure approaches 10%, it may suggest that the population's need for caesarean sections is met. Given the prevalent social inequalities, an optimum aggregate caesarean section rate at the state level may conceal inequities at the district level. Aggregated statistics conceal women who need but do not receive a caesarean section due to access or financial barriers by compensating with those who receive a caesarean section not medically indicated. Therefore, a more granular analysis of caesarean section rates at the district level is needed. (9)

For peer review only - http://bmjopen.bmj.com/site/about/guidelines.xhtml

In this paper, we report differences in caesarean section rates between population subgroups based on socioeconomic status across district wealth quintiles in Bihar. The study aimed to assess the geographical and economic inequalities in caesarean section rates between the districts of Bihar. In addition, the contributions of free government-funded public facilities and fee-for-service private facilities to population caesarean section rates are compared.

Methods

We conducted a secondary analysis of publicly available data from the National Family Health Survey (NFHS), a large-scale multi-round survey conducted in a representative sample of households throughout India. (7,10) The NFHS provides information on health and nutrition indicators at the sub-national level. The Government of India Ministry of Health and Family Welfare selected the International Institute for Population Sciences, Mumbai, as the nodal agency for the various rounds. The Woman's Schedule covered a wide variety of topics, including women's characteristics, marriage, fertility, contraception, children's immunisations and healthcare, nutrition, reproductive health, sexual behaviour, HIV/AIDS, women's empowerment and domestic violence, but not maternal deaths.

Data sources

For Bihar, the NFHS-5 data-collection was done in 2019–2020 from 35,834 households, which included 42,843 women aged 15–49 years. (7) The NFHS records whether a caesarean section was performed in a public or private facility. The wealth quintile data were available in the 2015–2016 NFHS-4 (8,10), and raw data for Bihar were provided by the nodal agency on request.

For peer review only - http://bmjopen.bmj.com/site/about/guidelines.xhtml

Study setting

Bihar, with a population in the 2011 census of approximately 104 million people (11), has a low GDP per capita (US$ 610) compared to other Indian states. (9,12) In total, 88% of the population of Bihar lives in rural areas. The state has the highest crude birth rate (26.1 per 1,000 population) in India, with one baby born every two seconds. (7) Bihar is divided into 38 administrative districts, 101 subdivisions and 534 blocks. Each district has a district (Sadar) hospital, and six districts also have one or more medical college hospitals.

Bihar's government healthcare system functions on three levels. At the primary level, sub-health centres and primary health centres function as the first point of contact. These primary facilities, providing basic emergency obstetric and newborn care (BEmONC) services, are mostly staffed by nurses, general physicians and alternative medicine practitioners but have no surgical capacity. At the secondary level, 149 first referral units (FRUs) function as first-level CEmONC units where caesarean sections are performed. These FRUs include district and sub-district hospitals (serving a population of 500,000–600,000) and community health centres (block-level hospitals), as per Indian Public Health Standards. (13) In addition, seven government and two autonomous medical colleges in Bihar are designated as tertiary centres, four of which are in the capital city of Patna (two government medical colleges and two autonomous medical colleges). Four medical colleges are located in North Bihar, four in Patna and only one in South Bihar. Medical colleges (though tertiary) fill a gap by also functioning as secondary health facilities because FRUs are poorly functional.

Of the mothers who delivered during the last five years in Bihar, 25.5% had at least four antenatal clinic visits and 89.5% were protected against neonatal tetanus. (7) Most

For peer review only

For peer review only - http://bmjopen.bmj.com/site/about/guidelines.xhtml

deliveries (76.2%) take place in an institution, with 56.9% in a public facility and 19.3% in a private facility.

According to the NFHS-4, (8) each household is assigned to a wealth quintile based on its household characteristics and scores established by the principal component analysis. (14). In Bihar, 80.2% of the population belongs to the two poorest quintiles, 11.4% to the middle quintile and only 8.36% to the two richest quintiles. (8) To compare districts, we combined the proportions of people in the two poorest quintiles.

Data analysis

Caesarean section rates and the proportion of institutional deliveries for each district were extracted from the NFHS-5, including the contributions by the public and private sectors. Since no optimum rate exists, we stratified the rates into <10%, 10–20% and >20% to measure equity in access to caesarean sections, from possible inadequate access to overuse. (15) The NFHS-4 and NFHS-5 caesarean section rates, respectively for 2015–2016 and 2019–2020, are presented in Figure 1 to demonstrate the geographical differences in change in rates between districts. Regression analysis was performed assessing the correlation between the proportion of the population in the two poorest quintiles and caesarean section rates. Both the caesarean section rate among all institutional births and the proportion of institutional deliveries in private and public health facilities were considered using the extrapolated caesarean section rate from the NFHS-5. (7)

For peer review only - http://bmjopen.bmj.com/site/about/guidelines.xhtml

Ethical considerations

Our study is based on the dataset from the NFHS-4 and NFHS-5 surveys with no identifiable information on the participants and can be freely accessed from the NFHS website. The ethical approval for the NFHS-4 and NFHS-5 surveys was obtained from the ethics review board of the International Institute for Population Sciences, Mumbai, India. These surveys were also reviewed and approved by the ICF International Review Board. Informed written consent for participation in this survey was obtained from the respondents during the survey. Each individual's approval was sought before the interview was conducted.

Patient and public involvement

Patients and the public were not involved in the development of the study design.

Results

Caesarean section rate trends across districts in Bihar

In Bihar, the caesarean section rate has increased from 6.2% in 2015 (NFHS-4) to 9.7% in 2019 (NFHS-5). Of the rate in 2019, 2.0% came from the public sector and 7.6% from the private sector. The NFHS-5 documented that the percentages of all live births delivered by caesarean section in public and private facilities were 3.6% and 39.6%, respectively.

Figure 1 shows the district-wise change in the rate of caesarean sections from 2015 to 2019. The number of districts with caesarean section rates below 10% decreased from 34 in 2015 to 21 in 2019. Using the 10% rate to evaluate access to caesarean sections in the 38 district populations, 14 districts were above 10% in 2019, three were at 10% and 21

For peer review only - http://bmjopen.bmj.com/site/about/guidelines.xhtml

were below 10%. Three districts (Jehanabad, Munger and Patna) had caesarean section rates above 20%.

The degree of change in caesarean section rates was uneven at the district level. Of the 38 districts, 28 demonstrated a remarkable increase of 5.9% in caesarean section rates in four years (from 5.5% in 2015 to 11.4% in 2019). However, against this trend was a 2.1% reduction in caesarean section rates in 10 districts (from 8.5% in 2015 to 6.5% in 2019).

Surrounding Patna are 10 adjacent districts within 86 kilometres or a two-hour driving time. In these districts, 77.4% of the population belongs to the two poorest wealth quintiles. Moving away from the capital city, we noted a decrease in the caesarean section rate to 11.3% (of which 9.3% were in private and 2.0% were in public facilities). The more remote districts are on average 192 kilometres away from the capital, or 5–6 hours transit. Of the population in these remote districts, 81.1% are in the two poorest quintiles, and the furthest from the capital had a further caesarean section rate drop to 9.7% (of which 7.5% were in private and 2.2% were in public facilities). Public facilities conducted proportionally more caesarean sections in districts with a larger proportion of people from poorer quintiles.

Figure 1: Caesarean section rate by district in 2015 and 2019

Comparison of caesarean section rate in the districts of Bihar in 2015 (NFHS-4) and 2019 (NFHS-5).

Among all institutional births at public facilities, the caesarean section rate markedly decreased from 8.2% in 2015 to 2.1% in 2019. Contrary to the overall rising trend of caesarean section rate, the number of government public facilities performing at least

For peer review only

For peer review only - http://bmjopen.bmj.com/site/about/guidelines.xhtml

10% caesarean sections among all deliveries dropped from eight in 2015 to zero in 2019. The districts with university teaching hospitals and medical colleges (Bhagalpur, Nalanda, Darbhanga, Gaya, Muzaffarpur and Patna) in addition to district hospitals had district-level caesarean section rates higher than 10%, except West Champaran (6.4%).

In contrast, the caesarean section rate increased in private facilities from 6.8% in 2015 to 8.0% in 2019. Only four districts in 2015 had a caesarean section rate of >10% in private facilities, which increased to nine districts in 2019.

Caesarean section rate trends by district wealth quintiles in Bihar

Districts with higher proportions of the two richest quintiles (Bhagalpur, Buxar, Nalanda, Patna and Rohtas) had the highest caesarean section rates among all institutional births, with 11.8% in private facilities and 2.7% in public health facilities. Patna, the capital district of Bihar (and with the wealthiest people in the state), had the highest caesarean section rate in private facilities (17.9%) and a higher than average rate in public health facilities (4.0%). Supaul is the poorest district in Bihar, with 94.1% of the population in the lowest two quintiles; of all deliveries, 1.8% and 1.9% were delivered by caesarean respectively in private and public facilities.

Figure 2 shows with decreasing wealth was a corresponding decrease in caesarean section rates (Figure 2). The negative correlation ($R^2 = 0.46$) between population wealth quintiles and caesarean section rates in private health facilities demonstrates that access to private caesarean sections dropped dramatically in poorer populations. However, caesarean section rates dropped in poorer populations even in public facilities, though the correlation was weak ($R^2 = 0.10$). Public health facilities function independent of the wealth status of the population and are proportionately more available for the poor, who have lesser access to private care.

For peer review only - http://bmjopen.bmj.com/site/about/guidelines.xhtml

Figure 2: Proportion of total deliveries by caesarean section in private and public sector by wealth status in the districts of Bihar in 2015 (NFHS-4) and 2019 (NFHS-5).

The x-axis represents the proportion of the population classified in the two poorest wealth quintiles. The y-axis represents the proportion of all deliveries in the district delivered by caesarean section. For each district in Bihar, the contribution of the private sector (blue circle) and the public sector (orange square), together with the total rate of caesarean sections, are shown with individual markers. For each category, a linear regression line is added, including the coefficient of correlation (R2 significant value between -1 to +1).

Institutional Delivery rate trends by district wealth quintiles in Bihar

The institutional delivery rate in Bihar increased from 63.8% in 2005 to 76.2% in 2019. Overall institutional births showed a negative linear correlation (R^2 = 0.43) with the wealth status of the population, and homebirths were still associated with poorer populations(Figure 3). Populations in poorer districts are more reliant on public facilities for institutional deliveries and birth preparedness. Figure 3 shows that with increasing poverty, institutional deliveries in private facilities decreased, with a negative linear correlation (R^2 = 0.64). Among the poorest two quintiles, the proportion of institutional deliveries in public facilities was higher than in private facilities. In the same group, the opposite was observed for caesarean sections, which are predominantly performed in the private sector.

Figure 3: Proportion of total deliveries in private and public institutions by wealth status in the districts of Bihar in 2015 (NFHS-4) and 2019 (NFHS-5).

For peer review only - http://bmjopen.bmj.com/site/about/guidelines.xhtml

Discussion

In Bihar, the caesarean section rate increased from 6.2% in 2015 to 9.7% in 2019. However, caesarean sections were not equally distributed, and districts with a larger proportion of poor people had a lower rate of institutional deliveries and a lower rate of caesarean section rates compared to districts with a higher proportion of people in the two richest quintiles. The contribution of the private sector to the overall caesarean section rate was high, but it was lower in the poorer districts, while the contribution of the public sector was approximately the same.

<u>Caesarean sections and the health system</u>

Caesarean section rates are driven by supply-side (such as resources within the health system, healthcare policy and strategies, health financing systems and perceptions of healthcare professionals) and demand-side (such as socioeconomic status, population preference and perceptions and trust in the health system) determinants. This study reinforces the importance of government maternal health programs in promoting institutional deliveries in resource-poor economies. The poor population chooses to deliver in public health facilities because they are affordable and incentivised. In the poorest regions, establishing private facilities is not profitable because the paying capacity of the population is low, and therefore the private option is simply not available. The scale-up of institutional deliveries has been possible due to extensive and prolonged training programs for auxiliary nurses and general nurse midwives, supported by task-shifted cadres of helpers in the labour delivery suite. (16)

For peer review only - http://bmjopen.bmj.com/site/about/guidelines.xhtml

Uneven caesarean section rates across geographical distribution

Bihar is divided into North and South Bihar, with marked variations in geography and health. (17) North Bihar is downstream of the seasonally flooding Himalayan rivers, whereas South Bihar is in the drought zone. In both areas, the availability and utilisation of health service facilities are driven by the socioeconomic status of the population and provider factors rather than physical access. (16) Geographical access to health facilities has improved in Bihar, with better roads, electricity and transportation over the past decade. (10) The eastern districts in Bihar are insecure workplaces for healthcare professionals due to political instability. Bihar has the lowest population-to-doctor ratio in the country, and the available doctors tend to choose urban areas for government service or private practice. (18)

The caesarean section rate varies across the districts of Bihar. We found that districts closer to the capital city of Patna had higher caesarean section rates. Other factors influencing the caesarean section rate were the number of active specialists, availability of private facilities and blood availability. All these factors decrease with distance from the capital. The caesarean section rates in Bihar display the entire spectrum from too little (inadequate access) to too much (overuse), all within one state. The powerful global acceptance of technological solutions and medicalising birth is set against the local traditional preference for vaginal delivery, regardless of educational status and across wealth quintiles in the Bihar population. The poorer population faces affordability and access barriers to caesarean sections. (18) Cultural beliefs and practices about vaginal birth, irrespective of wealth quintiles, make it the preferred mode of delivery over caesarean section. Performing a caesarean section is still viewed as a lack of competence of the provider to perform an unassisted or assisted vaginal delivery, but this may also simply reflect a lack of trust or fear of a surgical procedure. Research has shown that

For peer review only - http://bmjopen.bmj.com/site/about/guidelines.xhtml

demand for caesarean sections has been relatively uncommon in Bihar (16,18–21) due to cosmetic concerns, fear of pain during childbirth and sexual functioning concerns, foregrounded by the upper wealth quintiles from other nations (2) or other Indian states (19).

Distribution of caesarean section rates between public and private health facilities

Free-service public facilities are the first point of access for women with high-risk pregnancies in this predominantly poor state. However, while BEmONC services have been strengthened in public facilities, our findings show a quantitative reduction in CEmONC services available to the same women who chose to deliver in public facilities. While more than six out of 10 women delivered in public facilities, only around one in 28 women (3.6%) received a caesarean section in these public hospitals. Two in five children were delivered by caesarean section in private hospitals (39.6%). A proportionate decline occurred in the outputs and performance of public health facilities in Bihar over five years (2015–2019). Of 149 designated public FRUs, only 40 provide some CEmONC services, usually during office hours. The better-functioning public facilities, with significantly higher caesarean section rates, are attributed to the presence of medical colleges, teams of specialist doctors and greater urbanisation in those districts. (2) In the poorer-performing districts, low output is commonly attributed to inadequate infrastructure and staffing. However, rates remain low even in well-resourced public facilities with adequate infrastructure. (22) Our results show that caesarean section services in districts with the largest proportion of population in the poorest wealth quintiles have poor access to care in both public and private health facilities.

The supply-side provider barriers in public facilities are hesitancy to treat at-risk pregnant women, lack of clinical support for complications, administrative support for litigation,

For peer review only - http://bmjopen.bmj.com/site/about/guidelines.xhtml

and occasional episodes of violence against healthcare workers. Government doctors who refuse to perform caesarean sections in public facilities are willing to operate on the same high-risk women in private facilities. Professional social networks provide a safety net and allow for higher risk-taking in private facilities, though the infrastructure is better in public facilities. Financial incentives to providers via government schemes (Janani Shishu Suraksha Karyakram) with performance-based incentives for doctors have not increased the provision of caesarean sections at government facilities. (17) Women with high-risk pregnancies are referred to higher-level facilities (private facilities, medical colleges or tertiary facilities) for caesarean sections. (23) The demand-side patient barriers for low uptake of caesarean sections include user fees, sociocultural beliefs around the value of the procedure, a longer stay in the hospital and a trust deficit about the safety of caesarean sections. Costs in private hospitals are at least two to four times those in public hospitals. (7,24) The fee structure in the private sector is not regulated by the government, and the private sector is therefore free to decide user fees. Currently, the predominantly urban private sector in Bihar is still inadequate to provide for the needs of the 80% of the population in the two poorest wealth quintiles. Therefore public facilities need to be functional to provide free and affordable caesarean section services.

Caesarean section distribution across wealth quintiles and equitable solutions

Caesarean section is a life-saving operation that should be available to all women. The probable reasons for the inequity and unaffordability of healthcare services include insufficient public financing and consequent out-of-pocket expenditure to meet the high costs of caesarean section services. (25)

To address inequity in access, the Government of India launched a conditional cash-transfer scheme to promote institutional deliveries by providing free services to neonates

For peer review only - http://bmjopen.bmj.com/site/about/guidelines.xhtml

and pregnant women, aiming to encourage marginalised pregnant women to use free public health facilities. More recently, the world's largest health insurance scheme, Pradhan Mantri Jan Arogya Yojana (PM-JAY), was launched in 2018 to provide free services for the 500 million poor in India. Caesarean section is included in the PM-JAY maternal package to mitigate the impact of the catastrophic expenditure incurred. However, the overall uptake of PM-JAY reimbursement rates by private providers remains poor in the state. (26)

Lessons can be learned from other LMICs, such as Cambodia, Indonesia and Ghana, which have previously introduced policies to remove user fees for pregnant women. (2) Rwanda has set an example with its community-based health insurance scheme to reduce out-of-pocket payments for patients and a performance-based financing system to incentivise providers. (27) The key findings from our results – that although the overall caesarean rate has increased among the poor, the rates for the poorest subgroup still fall short – are also reflected by findings from Ghana and Rwanda. Like Ghana, Bihar exempts women from paying for delivery care and has a state policy of demand-side financing by incentivising institutional deliveries to reduce barriers to access to quality care in remote areas. Cambodia has identified and targeted the poor for user fee exemptions and health insurance provision through health equity funds. This scheme is similar to Indonesia's social health insurance program, *Jamkesmas*, for poor and near-poor people, which has been shown to improve access and reduce financial barriers. (28) In Bhutan, only small differences in caesarean section rates between wealth quintiles have been observed, with around one in eight of all deliveries by caesarean section. However, no privatisation of healthcare exists in this LMIC. (2,29) Like Bihar, Burundi demonstrates increasing inequalities and caesarean section disparities with improved access to maternal healthcare. (30)

For peer review only - http://bmjopen.bmj.com/site/about/guidelines.xhtml

Strengths and limitations

A strength of this study is its use of secondary data from India's largest and most reliable household-level survey data sources, the NFHS. It also provides insight on a more granular level into the distribution of caesarean sections on district level and its relation to economic status. Finally, the role of the public sector is an important element that was considered and essential for the understanding of the distribution of caesarean sections.

A limitation of this study is that economic status was retrieved from the NFHS-4 because the NFHS-5 had not yet released its wealth quintile data at the time of writing. In addition, no clear consensus exists on an exact optimum caesarean section rate for maximum impact on reducing maternal mortality and minimal risk for unnecessary interventions. Therefore, the cut-offs of 10% and 20% were pragmatically chosen based on the available evidence.

Conclusion

Aggregated state-level caesarean section rates conceal the inequity of adequate access to emergency obstetric care among the poorest population, which is compensated by the high levels of caesarean use in the richest and most advantaged subgroups. Using relevant equity stratifiers, population subgroups and specific strategies need to be identified to address the inequities by improving access to maternal and newborn health services in public health facilities. The role of the private sector in meeting the demand for caesarean sections for the population is strongly substantiated, but the concomitant out-of-pocket expenditure is debilitating and catastrophic.

Contributorship statement: Nobhojit Roy, Anita Gadgil and Alex van Duinen conceptualised and planned the research project. Minal Shukla, Monali Mohan, Juul

For peer review only - http://bmjopen.bmj.com/site/about/guidelines.xhtml

Bakker and Pranav Bhushan conducted the analysis. All contributed to the writing of the manuscript.

Acknowledgments: We thank Priti Patil for her input in the manuscript.

Competing interests: None of the authors have any competing interests.

Funding: It was a non-funded project.

Data sharing statement: Data are available in a public, open access repository, additionally, data can be shared by the corresponding author.

Ethics approval statement: This was an analysis of secondary data from open access and no primary data has been collected from human subjects.

References

1. Sobhy S, Arroyo-Manzano D, Murugesu N, Karthikeyan G, Kumar V, Kaur I, et al. Maternal and perinatal mortality and complications associated with caesarean section in low-income and middle-income countries: a systematic review and meta-analysis. Lancet [Internet]. 2019;393(10184):1973–82. Available from: http://dx.doi.org/10.1016/S0140-6736(18)32386-9

2. Boatin AA, Schlotheuber A, Betran AP, Moller AB, Barros AJD, Boerma T, et al. Within country inequalities in caesarean section rates: Observational study of 72 low and middle income countries. BMJ. 2018;360.

3. Molina G, Weiser TG, Lipsitz SR, Esquivel MM, Uribe-Leitz T, Azad T, et al. Relationship between cesarean delivery rate and maternal and neonatal mortality. JAMA - J Am Med Assoc. 2015;314(21):2263–70.

4. International Institute for Population Sciences. National Family Health Survey (NFHS-1) 1992-1993 India. Int Inst Popul Sci [Internet]. 1995. Available from:

For peer review only - http://bmjopen.bmj.com/site/about/guidelines.xhtml

http://rchiips.org/nfhs/india1.shtml

5. Sample Registration System: Special Bulletin on Maternal Mortality in India 2016-2018 [Internet]. New Delhi; 2020. Available from: https://censusindia.gov.in/vital_statistics/SRS_Bulletins/MMR%20Bulletin%202016-18.pdf

6. Meara JG, Leather AJM, Hagander L, Alkire BC, Alonso N, Ameh EA, et al. Global Surgery 2030: evidence and solutions for achieving health, welfare, and economic development. Lancet [Internet]. 2015;386(9993):569–624. Available from: http://www.ncbi.nlm.nih.gov/pubmed/25924834?otool=karolib&tool=karolinska

7. International Institute for Population Sciences. National Family Health Survey-5 (Bihar State Report) 2019-2020 [Internet]. Mumbai; 2020. Available from: http://rchiips.org/nfhs/NFHS-5_FCTS/FactSheet_BR.pdf

8. International Institute for Population Sciences. National Family Health Survey (NFHS-4) 2015-16 India. Int Inst Popul Sci ICF [Internet]. 2017;1–192. Available from: http://rchiips.org/NFHS/NFHS-4Reports/India.pdf

9. Guilmoto CZ, Dumont A. Trends, Regional Variations, and Socioeconomic Disparities in Cesarean Births in India, 2010-2016. JAMA Netw open. 2019;2(3):e190526.

10. International Institute for Population Sciences. National Family Health Survey-4 (Bihar State Report) 2015-2016 [Internet]. Mumbai; 2020. Available from: http://rchiips.org/nfhs/NFHS-4Reports/Bihar.pdf

11. Census Organization of India. Census of India 2011: Bihar [Internet]. [cited 2020 Jul 20]. Available from: http://www.census2011.co.in/census/state/bihar.html

12. Srivastava S, Chaurasia H, Kumar Singh KJ, Chaudhary P. Exploring the spatial

For peer review only

For peer review only - http://bmjopen.bmj.com/site/about/guidelines.xhtml

patterns of cesarean section delivery in India: Evidence from National Family Health Survey-4. Clin Epidemiol Glob Heal [Internet]. 2019;(September):1–9. Available from: https://doi.org/10.1016/j.cegh.2019.09.012

13. Directorate General of Health Services Ministry of Health & Family Welfare Government of India. Indian Public Health Standards (IPHS) Guidelines for Community Health Centres [Internet]. New Delhi; 2012. Available from: http://nhm.gov.in/images/pdf/guidelines/iphs/iphs-revised-guidlines-2012/community-health-centres.pdf

14. Rutstein SO. Steps to constructing the new DHS Wealth Index [Internet]. 2014. Available from: https://dhsprogram.com/programming/wealth index/Steps_to_constructing_the_new_DHS_Wealth_Index.pdf

15. Betran AP, Torloni MR, Zhang JJ, Gülmezoglu AM. WHO statement on caesarean section rates. BJOG An Int J Obstet Gynaecol. 2016;123(5):667–70.

16. Creanga AA, Srikantiah S, Mahapatra T, Das A, Sonthalia S, Ranjan Moharana P, et al. Statewide implementation of a quality improvement initiative for reproductive, maternal, newborn and child health and nutrition in Bihar, India. J Glob Health. 2020;10(2):1–13.

17. Salazar M, Vora K, Annerstedt KS, De Costa A. Caesarean sections in the in the context of the Chiranjeevi Yojana public private partnership program to promote institutional birth in Gujarat, India; Does the embedded disincentive for caesarean section work? Int J Equity Health. 2019;18(1):1–8.

18. Kaur J, Franzen SRP, Newton-Lewis T, Murphy G. Readiness of public health facilities to provide quality maternal and newborn care across the state of Bihar, India: A cross-

For peer review only

sectional study of district hospitals and primary health centres. BMJ Open. 2019;9(7):1–10.

19. Vora KS, Yasobant S, Sengupta R, De Costa A, Upadhyay A, Mavalankar D V. Options for optimal coverage of free C section services for poor mothers in indian state of Gujarat: Location allocation analysis using GIS. PLoS One. 2015;10(9):1–16.

20. Office of the Registrar General & Census Commissioner. Annual Health Survey:2011-12 Fact Sheet Bihar. 2012;1–176. Available from: www.censusindia.gov.in

21. Dhirar N, Kumar R, Dudeja S. Upward trend of caesarean sections in India: Incision over precision. Vol. 31, National Medical Journal of India. 2018. p. 316–7.

22. National Health Mission. 12th Common Review Mission [Internet]. New Delhi; 2019 [cited 2021 May 2]. Available from: http://nhsrcindia.org/sites/default/files/12th Common Review Mission-Report 2018.pdf

23. Bhatia M, Banerjee K, Dixit P, Dwivedi LK. Assessment of Variation in Cesarean Delivery Rates Between Public and Private Health Facilities in India From 2005 to 2016. JAMA Netw open. 2020;3(8):e2015022.

24. Radhakrishnan A, Vasanthakumari K, PK B. Increasing Trend of Caesarean Rates in India: Evidence from NFHS-4. J Med Sci Clin Res [Internet]. 2017 Aug 7;5(8):26167–76. Available from: http://jmscr.igmpublication.org/v5-i8/31 jmscr.pdf

25. Balarajan Y, Selvaraj S, Subramanian S. Health care and equity in India. Lancet [Internet]. 2011 Feb;377(9764):505–15. Available from: https://linkinghub.elsevier.com/retrieve/pii/S0140673610618946

26. National Health Authority. Report on Top Utilized Private Hospitals - Ayushman Bharat- Pradhan Mantri Jan Arogya Yojana (AB PM-JAY) [Internet]. New Delhi;

For peer review only

For peer review only - http://bmjopen.bmj.com/site/about/guidelines.xhtml

2020. Available from: https://pmjay.gov.in/sites/default/files/2019-10/Report on top utilized pvt hospitals_Final.pdf

27. Gibbons L, Belizán J, Lauer J, Betrán A, Merialdi M, Althabe F. The Global Numbers and Costs of Additionally Needed and Unnecessary Caesarean Sections Performed per Year: Overuse as a Barrier to Universal Coverage HEALTH SYSTEMS FINANCING [Internet]. Vol. 30, World Health Report 2010. 2010 Jan.

28. Zahroh RI, Disney G, Betrán AP, Bohren MA. Trends and sociodemographic inequalities in the use of caesarean section in Indonesia, 1987-2017. BMJ Glob Heal. 2020;5(12).

29. Hanson C, Betrán AP, Opondo C, Mkumbo E, Manzi F, Mbaruku G, et al. Trends in caesarean section rates between 2007 and 2013 in obstetric risk groups inspired by the Robson classification: results from population-based surveys in a low-resource setting. BJOG An Int J Obstet Gynaecol. 2019;126(6):690–700.

30. Yaya S, Zegeye B, Idriss-Wheeler D, Shibre G. Inequalities in caesarean section in Burundi: Evidence from the Burundi Demographic and Health Surveys (2010-2016). BMC Health Serv Res. 2020;20(1):1–8.

For peer review only

For peer review only - http://bmjopen.bmj.com/site/about/guidelines.xhtml

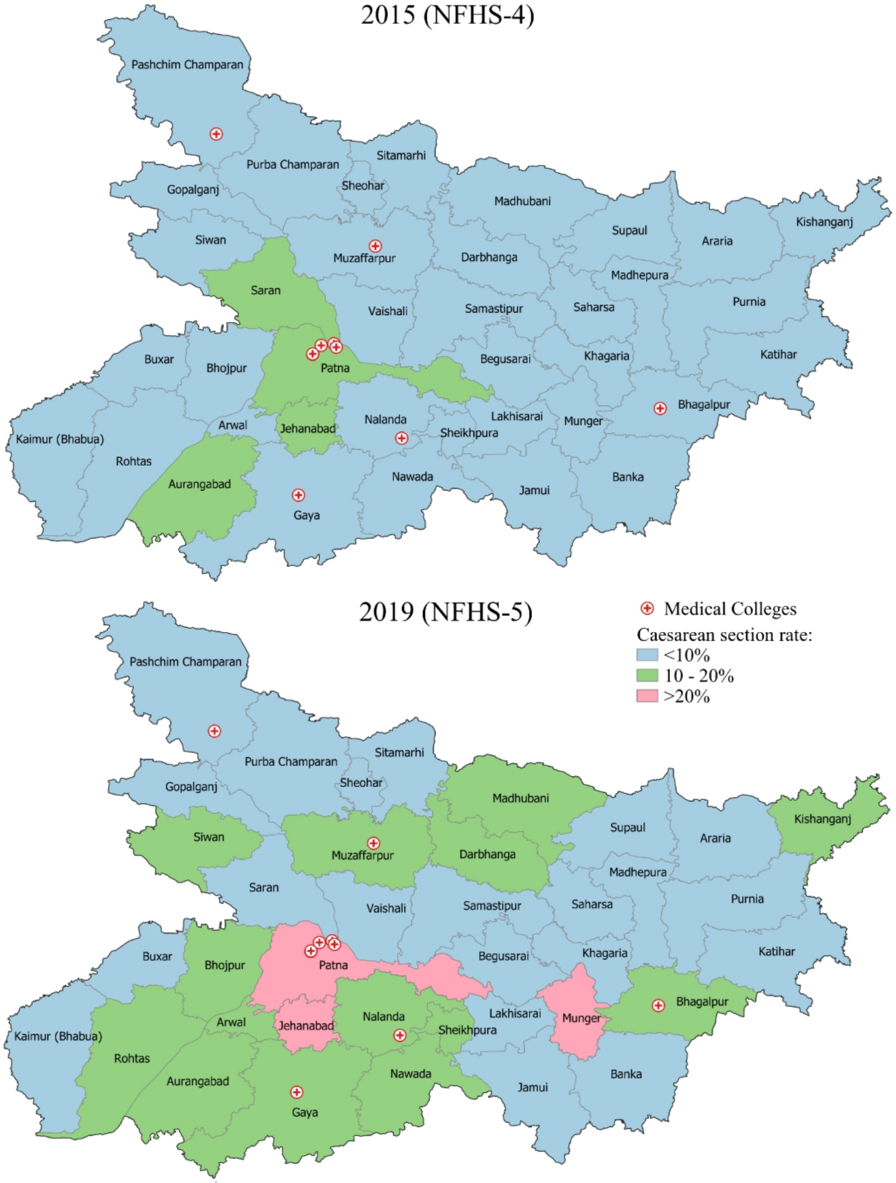

Comparison of caesarean section rate in the districts of Bihar in 2015 (NFHS-4) and 2019 (NFHS-5).

209x280mm (300 x 300 DPI)

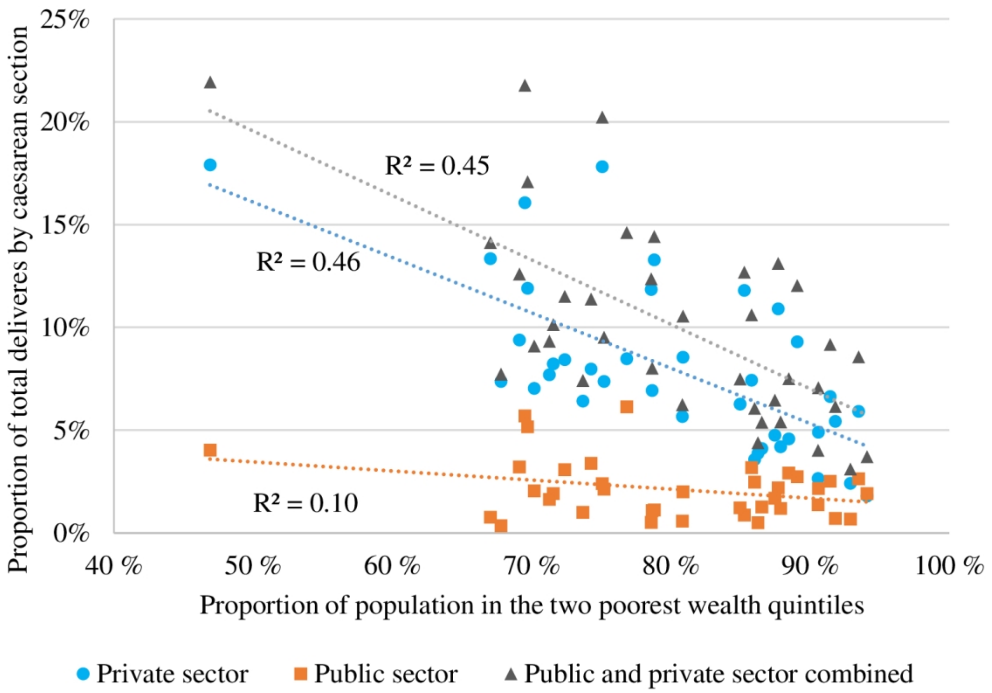

The x-axis represents the proportion of the population classified in the two poorest wealth quintiles. The y-axis represents the proportion of all deliveries in the district delivered by caesarean section. For each district in Bihar, the contribution of the private sector (blue circle) and the public sector (orange square), together with the total rate of caesarean sections, are shown with individual markers. For each category, a trendline and the R^2 result are provided.

132x93mm (300 x 300 DPI)

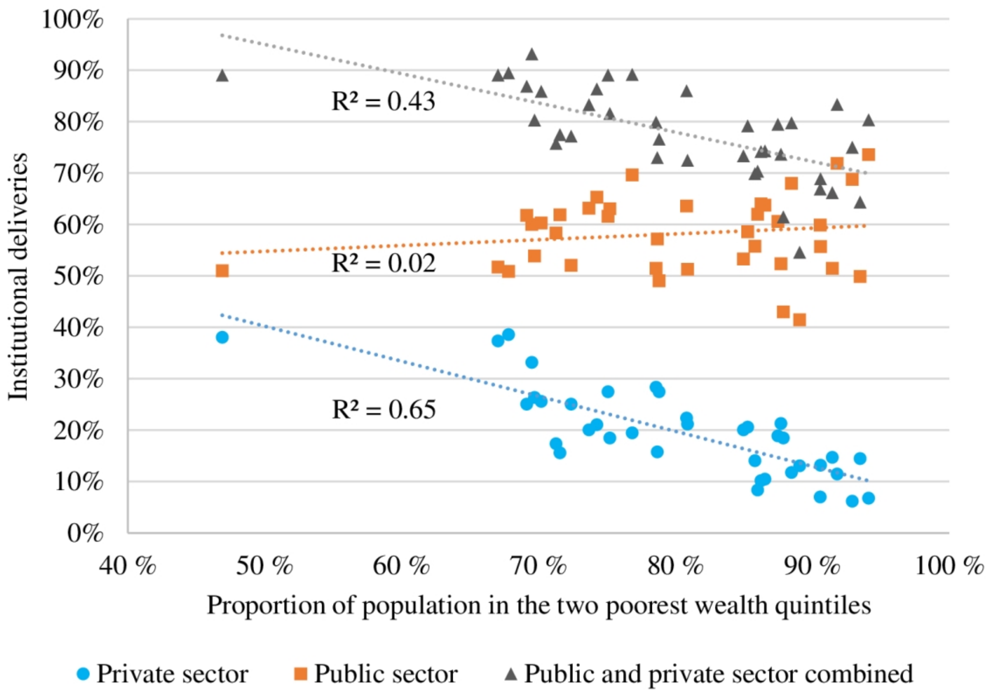

The x-axis represents the proportion of the population classified in the two poorest wealth quintiles. The y-axis represents the proportion of all deliveries in the district born in an institution. For each district in Bihar, the contribution of the private sector (blue circle) and the public sector (orange square), together with the total institutional delivery rate are shown with individual markers. For each category, a trendline and the R^2 result are provided.

132x93mm (300 x 300 DPI)

STROBE CHECKLIST			
**Section/topic**	**Item number**	**Page Number**	**Recommendation**
**Title and abstract**	1	1-3	**Title**: Assessing geographical and economic inequalities in caesarean section rates between the districts of Bihar, India: A secondary analysis of the National Family Health Survey. **Abstract:** In Bihar, one of the most populous and poorest states in India, caesarean sections have increased over the last decade. By retrospective secondary data analysis based on open-source national datasets from the 2015 and 2019 National Family Health Surveys we found there is marked inequalities exist in access to caesarean sections. The public sector needs to be strengthened to improve access to obstetric services for those who need it most.
**Introduction**	2	4	
Background/rationale	3	NA	In India caesarean section rates are now almost at par with the global figures. In Bihar, which is most populous and poorest states in India caesarean sections increased over the last decade. Though state level aggregate caesarean rates in Bihar have risen to be near global figures, it conceals the inequities at the district level and vary as per economic status of the communities.
Objectives	4	5	The study aimed to assess the geographical and economic inequalities in caesarean section rates between the districts of Bihar. In addition, the contributions of free government-funded public facilities and fee-for-

STROBE CHECKLIST			
**Section/topic**	**Item number**	**Page Number**	**Recommendation**
			service private facilities to population caesarean section rates are compared.
**Methods**		5	
Study design	5	5	It is a Cross sectional study of secondary data study based on open source national datasets from 'National Family Health Surveys' of last two rounds in India (NFHS-4 (2015-16) and NFHS-5 (2019-20).
Setting	6	6	Study setting is the public health facilities and private facilities of northern state of India Bihar. It is a secondary data analysis from National Family Health Survey.
Participants	7	5	Population covered in the two rounds of national survey from Bihar, India.
Variables	8	NA	Total caesarean section rates, caesarean section rates in Private facilities and C section rates in public facilities in the districts of Bihar as available from the National survey . Wealth quintile data of the population covered in Bihar

STROBE CHECKLIST			
**Section/topic**	**Item number**	**Page Number**	**Recommendation**
Data sources/measurement	9*	5	This retrospective secondary data analysis was based on open source national datasets from 'National Family Health Surveys' of last two rounds.
Bias	10	NA	The Government of India Ministry of Health and Family Welfare selected the International Institute for Population Sciences, Mumbai, as the nodal agency for the various rounds of this survey. Sample from Bihar gathered from 35,834 households, 42,483 women, and 4,897 men.
Study size	11	5	Entire data set collected from Bihar regarding caesarean section and distribution of population across wealth quintiles was used. No sample drawn from this
Quantitative variables	12	6	According to the NFHS-4 the Indian population is divided into wealth quintiles. Based on this division, the proportion in each of these wealth quintiles for each district has been published.
Statistical methods	13	7	Statistical methods used is regression analysis. Population sub-groups falling under two poorest wealth quintile pregnant women sought for delivery services in public and private health facilities.

**STROBE CHECKLIST**			
**Section/topic**	**Item number**	**Page Number**	**Recommendation**
Results	14	8	
Participants	15*	5	Entire data set collected from Bihar regarding caesarean section and distribution of population across wealth quintiles was used. No sample drawn from this
Descriptive data	16*	NA	Not applicable
Outcome data	17*	8	Distribution of C sections across various wealth quintiles at districts in Bihar. Distribution of caesarean sections done in private and public facilities across wealth quintiles
Main results	18	8-12	Caesarean section rate increased from 6.20% in 2015 to 9.70% in 2019 in Bihar. Districts with a lower proportion of poor had higher caesarean section rates (R2=0.45) among all institutional births with 10.32% among them in private and 2.89% in public facilities. Access to private caesarean sections decreased (R2=0.46) for districts with poorer populations.
Other analyses	19	NA	Not applicable.

STROBE CHECKLIST			
**Section/topic**	**Item number**	**Page Number**	**Recommendation**
Discussion	20	8-16	Across the LMIC inequality in health care access is evident because of various reasons like geography, economic conditions etc. Population sub group in poor wealth quintile agonize for access of services like institutional delivery and caesarean section in public as well as private health facilities. However government made many policy level decisions to reduce the hurdles for poor.
Key results	21	12	Caesarean section rate increased from 6.20% in 2015 to 9.70% in 2019 in Bihar. Districts with a lower proportion of poor had higher caesarean section rates (R2=0.45) among all institutional births with 10.32% among them in private and 2.89% in public facilities. Access to private caesarean sections decreased (R2=0.46) for districts with poorer populations.
Limitations	22	17	A limitation of this study is that economic status was retrieved from the NFHS-4 because the NFHS-5 had not yet released its wealth quintile data at the time of writing. In addition, no clear consensus exists on an exact optimum caesarean section rate for maximum impact on reducing maternal mortality and minimal risk for unnecessary interventions. Therefore, the cut-offs of 10% and 20% were pragmatically chosen based on the available evidence.

STROBE CHECKLIST			
**Section/topic**	**Item number**	**Page Number**	**Recommendation**
Interpretation	23	8-16	There are marked inequalities in access to caesarean sections. Public sector need to strengthen to maintain access to obstetric services for those that need it from poorer wealth quintiles
Generalizability	24	NA	Not applicable.
Other information	25		
Funding	26	18	Non- funded research.

w only
